# Enhancing antibody affinity through experimental sampling of non-deleterious CDR mutations predicted by machine learning

**DOI:** 10.1038/s42004-023-01037-7

**Published:** 2023-11-09

**Authors:** Thomas Clark, Vidya Subramanian, Akila Jayaraman, Emmett Fitzpatrick, Ranjani Gopal, Niharika Pentakota, Troy Rurak, Shweta Anand, Alexander Viglione, Rahul Raman, Kannan Tharakaraman, Ram Sasisekharan

**Affiliations:** 1 Altus Enterprises, 900 Middlesex Turnpike, Billerica, MA USA; 2 Department of Biological Engineering, Koch Institute for Integrative Cancer Research, Massachusetts Institute of Technology, 77 Massachusetts Avenue, Cambridge, MA 02139 USA

**Keywords:** Antibody therapy, Protein design, Computational chemistry

## Abstract

The application of machine learning (ML) models to optimize antibody affinity to an antigen is gaining prominence. Unfortunately, the small and biased nature of the publicly available antibody-antigen interaction datasets makes it challenging to build an ML model that can accurately predict binding affinity changes due to mutations (ΔΔ*G*). Recognizing these inherent limitations, we reformulated the problem to ask whether an ML model capable of classifying deleterious vs non-deleterious mutations can guide antibody affinity maturation in a practical setting. To test this hypothesis, we developed a Random Forest classifier (Antibody Random Forest Classifier or AbRFC) with expert-guided features and integrated it into a computational-experimental workflow. AbRFC effectively predicted non-deleterious mutations on an in-house validation dataset that is free of biases seen in the publicly available training datasets. Furthermore, experimental screening of a limited number of predictions from the model (<10^2 designs) identified affinity-enhancing mutations in two unrelated SARS-CoV-2 antibodies, resulting in constructs with up to 1000-fold increased binding to the SARS-COV-2 RBD. Our findings indicate that accurate prediction and screening of non-deleterious mutations using machine learning offers a powerful approach to improving antibody affinity.

## Abstract (web summary)

The endeavor to harness machine learning to improve antibody-antigen interaction is not only significant but also fraught with challenges due to the limitations of the publicly available datasets. Here, the authors introduce an Antibody Random Forest Classifier that classifies mutations as either deleterious or non-deleterious based on physicochemical features, which enabled the identification of affinity-enhancing mutations in SARS-CoV-2 antibodies with an up to 1000-fold increased binding affinity.

## Introduction

Engineering antibodies for clinical development has been a constantly evolving field and there has always been tremendous interest in developing computational approaches to optimize antibody properties, especially the affinity of an antibody to a target antigen. These approaches typically involve prediction of the effect of one or more amino acid (AA) substitutions on antibody-antigen affinity in silico followed by experimental screening to validate the predictions. The in silico prediction of AA substitutions to optimize antibody affinity employs three-dimensional structural models of antibody-antigen complexes. Over the past decade, the in silico prediction tools have evolved from energy-based methods to statistical metrics derived from available three-dimensional structural data to machine learning (ML) approaches capable of learning model parameters from experimental and structural datasets.

More recently, fueled by the success of AlphaFold^1^, high-capacity (e.g., involving large numbers of model parameters) deep learning (DL) models have become increasingly popular for solving a variety of protein design problems, including antibody affinity enhancement. Several DL methods have been developed for predicting affinity enhancing mutations in antibodies^2–10^. Combining yeast or phage display libraries with sequence-based DL models has resulted in an efficient optimization of antibody affinity and clinical developability^2–6^. In a different approach using big sequence data sets, large language models (LLMs) pre-trained on the vast protein and antibody sequence space have been successfully employed in increasing the affinity of several anti-viral antibodies without requirement of three-dimensional structural models or antigen-specific context^7^. To incorporate structural information, studies have used publicly available data (e.g., SKEMPIV2^11^) that combines the measured effect of interface mutations with available crystal structures^8,9^. A recent method combining an ensemble of models including a graph neural network (GNN) trained on SKEMPIV2 with iterative experimental optimization was able to increase the affinity of an anti-SARS-COV-2 WT RBD antibody by 50-fold^10^.

The recent prominence gained by DL methods in antibody engineering has led to an overwhelming trend of abandoning expert-engineered features designed for small datasets. While this trend has gained popularity, there are important limitations to developing DL models as generalized methods for affinity enhancement in practical or translational antibody engineering. The large number of parameters that allow DL models to learn complex relationships from data also make them susceptible to overfitting. The successes of using DL models with display libraries avoid overfitting by requiring a large problem-specific experimental design space (10^4^–10^8^ designs) for model training. Pretrained LLMs, on the other hand, avoid overfitting by forgoing antigen-specific training entirely. However, this may limit their applicability in cases where enhancing Ab-Ag contacts is required, as evidenced by the LLM-based method’s inability to enhance the affinity of the S309 antibody towards the SARS-COV-2 Omicron variant despite its success enhancing affinity of other antiviral antibodies^7^. Given the limited number and types of mutations sampled in SKEMPIV2 (728 Ab/Ag single point mutations in 51 PDBs, 54% Ala-Scanning) that is used to train GNN antibody-specific models, the robust performance of these models to consistently optimize affinity across different antibodies using a small experimental screen size remains to be evaluated. Therefore, due to the complexity of antibody-antigen interactions and the limitations in current publicly available datasets, a need remains for establishing a robust ML model that can be integrated into a computational-experimental workflow.

Herein, we describe the use of data-driven model design and expert-engineered features that we have developed successfully over the years to optimize antibody binding affinity and specificity^12–16^ to build a practically usable ML model capable of selecting affinity enhancing mutations when integrated into an experimental workflow. Specifically, we address the limitations of the SKEMPIV2 dataset by training a Random Forest classifier, called Antibody Random Forest Classifier, to distinguish deleterious from non-deleterious mutations, maximally leveraging the information content in alanine mutations (ALA-scan) heavy data. This is distinct from the typically studied regression task of trying to predict the binding affinity upon mutation (ΔΔ*G*) directly, which was recently shown to be susceptible to overfitting on this dataset^17^. We selected the Random Forest architecture because properties such as ensemble learning, bootstrap sampling, feature randomization, and regularization help avoid or at least reduce overfitting. We assess Antibody Random Forest Classifier (AbRFC), along with the GNN and LLM-based models on an in-house validation dataset comprising diverse CDR mutations, finding that AbRFC outperforms the other models on this dataset.

To test AbRFC in a real-world scenario, we show that it can discover affinity-enhancing mutations when integrated into an experimental workflow. Using mutations predicted by AbRFC, we affinity enhanced two distinct starting template antibodies that had lost affinity to the Omicron variant. Given that these template antibodies target distinct epitope surfaces on SARS-COV-2, using them as test cases permitted us to assess the applicability of our model across different antibody-epitope interactions. For each antibody, we use two rounds of wet lab screening with less than 100 designs per round. The engineered antibodies show up to >1000-fold improved affinity compared to the corresponding template mAbs against various Omicron subvariants BA.1, BA.2, and BA.4/5. This study highlights the value of employing tree-based ML methods with expert-guided feature engineering and an appropriate use of training and validation datasets for achieving affinity enhancement across distinct antibody-epitope interactions with only two rounds of experimental screening of less than 100 constructs.

## Results

**Fig. 1:**
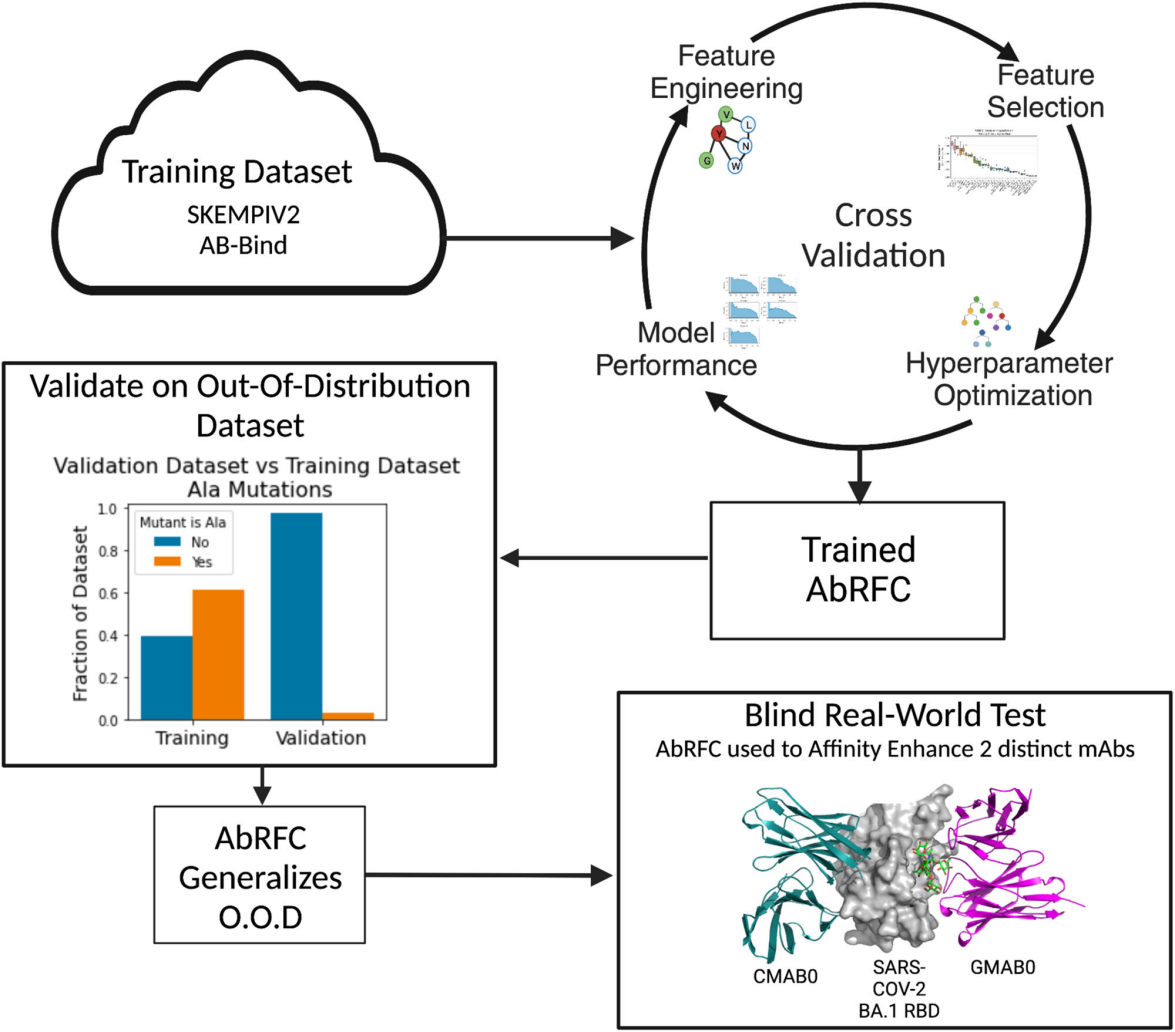
The workflow for developing and testing AbRFC. Public training data was used for model development with 5-fold cross-validation. Next, AbRFC was tested on an OOD validation dataset to assess the extent to which it could generalize beyond the training data. Finally, to show its practical utility, AbRFC was integrated into a computational-experimental workflow to affinity enhance two distinct anti-RBD templates with reduced affinity towards SARS-COV-2 BA.1 RBD.

The development and testing of the ML-model, AbRFC, involved the following key steps (illustrated in Fig. 1). First, we assessed the best modeling approach to capture the information in the available public training dataset. Next we engineered features guided by our past successes in optimizing antibody-binding affinity^12–16^. We used 5-fold cross-validation to optimize hyperparameters and increase the regularization of our model. Given that the training data was biased towards mutations to alanine, we tested performance of AbRFC on an out-of-distribution (OOD) validation dataset. After establishing the ability of AbRFC to generalize to the OOD dataset, we experimentally sampled non-deleterious mutations predicted by AbRFC to affinity enhance two distinct anti-SARS-COV-2 receptor-binding domain (RBD) template antibodies with reduced affinity towards SARS-COV-2 BA.1 RBD.

### Assessing the optimal use of the training data

**Fig. 2:**
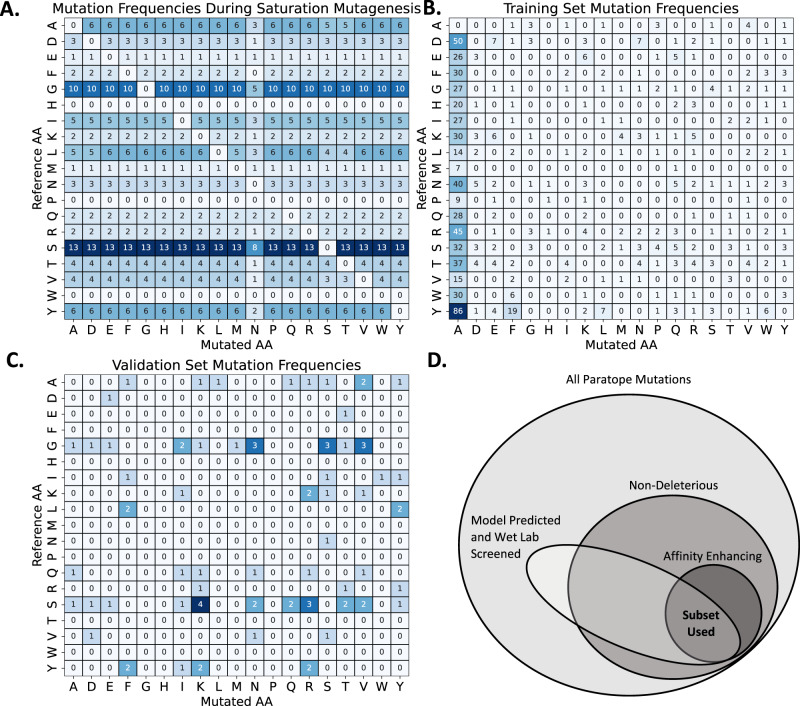
Frequency of mutations scored by a ML model in practice differs from training set. **A** In a real-world test scenario, mutation candidates are generated by in silico saturation mutagenesis. The AA counts depicted here are generated using saturation mutagenesis of on the PDB associated with the validation dataset. The mutant AA is uniformly distributed across the Reference AA according to the Reference AA paratope frequencies, which are typically enriched in Y, S, and G as seen here. **B** Training set mutations are dominated by Ala scanning (first column). **C** The validation set represents the mutations selected by a protein engineering expert to be screened in an independent affinity enhancing campaign (Supplementary Table 1). This mutation set is enhanced in mutations to Lys, Phe, and Tyr, and contains only three mutations to Ala. **D** The filtering hypothesis: given the OOD nature of the test distribution relative to the train distribution, we propose that the best use of the training data is to train a model to predict non-deleterious mutations. The filtering hypothesis proposes that this is sufficient to identify affinity enhancing mutations in a real-world test setting.

Given that SKEMPIV2 was the main public database comprising data on the impact of mutations on antibody binding affinity we sought to assess which “training data” from SKEMPIV2 (Methods) would recapitulate mutations seen during model application. To select mutations in real-world applications, we planned to use our model to score mutations generated by in silico saturation mutagenesis on the antibody paratope (Methods). Therefore, an ideal training dataset would contain a similar distribution of mutations as those seen during in silico saturation mutagenesis (Fig. 2A), where the mutant AA is distributed uniformly across the reference AA according to the reference AA paratope frequencies.

However, 61% of the mutations in the training dataset are to Alanine (Fig. 2B). Aromatic-to-aromatic and charged-to-charged mutations account for another 10% of the dataset, leaving 266 total observations for the remaining 298 possible reference-mutant pairs, or less than one sample per pair. Importantly, when we examined the mutant pairs that would be scored during a typical in silico saturation mutagenesis, we noticed that 43% of the pairs that the model would be asked to score were never seen in the training set. The observation that our model would have to generalize out of the distribution (OOD) of the training set led us to two critical design decisions.

Firstly, we formulated the filtering hypothesis (Fig. 2D). We reasoned that given much of the training set contains Ala mutations, which are used for hot-spot detection, it would be most valuable for training a model to classify deleterious and non-deleterious mutations. In the context of general protein-protein interactions, hotspots have been defined as residues with change in free energy on binding $\left|\Delta \Delta G\right| \, > \, 2{kcal}/{mol}$ (20% of training data), with residues having |$\Delta \Delta {G|}$ < 0.4 (34% of training data) as non-hotspots^18^. To avoid an imbalanced dataset and because we reasoned even small negative impacts may be detrimental to enhancing affinity, we used a cutoff of $\Delta \Delta G\ge -0.2$ to classify a mutation as non-deleterious, where −0.2 was selected as described in “Methods”. We hypothesized that the overlap of non-deleterious and affinity enhancing mutations was sufficiently large (Fig. 2D) to identify affinity enhancing mutations in under 100 wet lab screens if we filtered out the deleterious mutations.

The second design decision was to acknowledge that it would be necessary to assess the performance of our model on an OOD validation dataset. To this end, we used an in-house dataset of 79 point mutations generated by a protein engineering expert using structural information associated with relative binding values (Supplementary Table 1). This dataset is enriched in patterns that the expert has learned to apply in a residue-specific manner—for example in this instance frequently mutating L to F and Y and S to R and K (Fig. 2C) and therefore represents a distinct distribution of mutations relative to the training dataset. Using these two main design decisions, we built the AbRFC using the training dataset.

### Building AbRFC

Given the small and Ala-scan heavy nature of the training set, we reasoned that using previously validated metrics would decrease the likelihood of overfitting. Unlike features learned by neural networks directly from the training data, the previously validated metrics including Amino Acid Interface (AIF) score^12^, Significant Interaction Network (SIN) score^19^, and Rosetta energy terms^20^ are biased to encode information that experts anticipate will generalize across datasets. Because tree-based models are known to outperform neural networks on tabular datasets^21^, we selected a Random Forest Classifier to fit the extracted feature metrics to the labeled data in the training set. To assess model performance, we selected the area under the Precision-Recall curve (PR AUC) due to its robustness with respect to class imbalance. The PR AUC measures the tradeoff between precision and recall as the threshold model score to classify a mutation as non-deleterious varies, and PR AUC is a global metric that considers model performance at all thresholds. We used the predicted non-deleterious class probability from the Random Forest as the model score (Methods). With this performance metric and scoring function, we implemented 5-fold cross-validation on the training set (Methods) to perform feature engineering, feature selection, and hyperparameter optimization.

We engineered features by applying in silico metrices to two PDB structures, one with the reference AA and one with the mutant AA (Methods). The metric value for the mutant complex was then subtracted from the reference complex to form the feature value fed to AbRFC. To capture both local and global effects of a mutation, in addition to residue-specific features like $\Delta {{{{{\rm{AIF}}}}}}={{{{{\rm{AIF}}}}}}\left({{{{{{\rm{AA}}}}}}}_{{{{{{\rm{reference}}}}}}}\right)-{{{{{\rm{AIF}}}}}}\left({{{{{{\rm{AA}}}}}}}_{{{{{{\rm{mutant}}}}}}}\right)$, neighborhood and global complex features were calculated by summing the relevant metrices across the mutant residue’s neighbors or the full complex (Methods). After extracting 62 features, 50% of the features were pruned according to spearman’s rank correlation during cross-validation, leaving 31 features that were used in the final model (Supplementary Table 2).

**Fig. 3:**
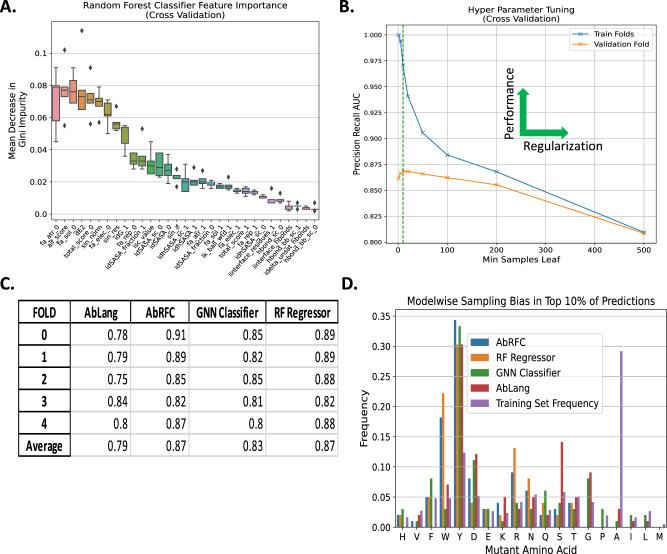
Analysis of AbRFC performance using cross-validation. **A** Feature Importance calculated by decrease in Gini Impurity (Methods) during cross-validation highlight the importance of previously derived statistical metrices including the AIF score and SIN scores in addition to Rosetta energy-based features. **B** The hyperparameter Min Samples Leaf was optimized during cross-validation. As this parameter is increased, regularization increases reducing the chance of overfitting, but limiting the function space expressible by the model given the training data. **C** Precision-Recall AUC was used to benchmark AbRFC against alternative models due to the imbalanced nature of the training set. The TREE-based models outperform the GNN and LLM models on this training set. **D** All the models exhibit difference amino acid preferences even though the three structural models were trained on the same data.

We observed the highest performing features included local and global scores and comprised both Rosetta energetics and previously validated metrices such as AIF and SIN (Fig. 3A). There is a 35% decrease in feature importance score between the 9th and 10th ranked features (Fig. 3A), suggesting that features ranked higher than this provide the majority of information to the model. These top features include both local terms such as the Rosetta-calculated Lennard-Jones attractive atomic potential between atoms in the mutated residue and surrounding residues (fa_atr_0), along with global scores such as the SIN normalization constant (sin_norm). Although feature selection can mitigate overfitting, we anticipated fitting hyperparameters would further improve the model’s performance.

Regularization is a critical ML method for reducing a model’s potential for overfitting by constraining the set of functions a model can approximate. In Random Forest models such as AbRFC, this is accomplished by specifying hyperparameters that limit the model capacity. Multiple hyperparameters were searched by comparing validation and training set performance during cross-validation (**Methods**). We found that min_samples_leaf, which requires a minimum number of samples to be present in leaf (decision) nodes, effectively regularized the model. Validation performance was maximized at min_samples_leaf ≈ 10 (Fig. 3B), which is a surprisingly large number given that there are only ~1200 examples in the training subset of each fold. This validated the need for extensive regularization given the nature of the training set, even when assessing within distribution performance during cross-validation.

### Cross-validation performance

After finalizing the AbRFC model, we compared the performance of this model with other model architectures. To determine the effect of our engineered features and model architecture choice, we included a GNN Classifier using the architecture from ref. ^10^ modified for the same binary task as AbRFC (Supplementary Methods). To assess our choice of classification over regression, we trained a Random Forest Regressor (RF Regressor) with identical hyperparameters to AbRFC to predict ΔΔ*G*. Finally, since LLMs have shown promising results on similar tasks, we also used an antibody-specific pretrained LLM (AbLang^22^). Continuous scores from each model were used to assess performance according to the PR AUC metric (Supplementary Fig. 1). Both RF models outperform the GNN classifier and AbLang (Fig. 3C). The GNN classifier is clearly consistently overfitting the data, with SoftMax scores approaching 0 or 1, while AbLang performs better than random despite the absence of training and lack of antigen information.

Interestingly, according to the PR AUC metric, the RF Regressor performs as well as AbRFC despite being trained on a regression task and evaluated on a classification task (Fig. 3C). Additionally, the average spearman *ρ* of AbRFC and the RF Regressor scores across 5 folds is .88. This suggested that AbRFC and the RF Regressor would pick equivalent mutations in practice. However, when we simulated the extent to which the same mutations would be picked by both models in practice (e.g., by taking the top 10%, 20%, or 30% of mutations), we found that only ~50% of the mutations picked by both models at each of these quantile cutoffs overlapped (Supplementary Table 3). These differences suggested that performance metrics on public datasets do not capture all the information regarding how models will select mutations when applied in antibody engineering use-cases.

To further assess the differences that would appear in practice, we calculated the AA frequencies in the top 10% of predictions for each model. All the models, including AbLang, prefer Y substitutions the most, which is unsurprising given its importance in antibody paratopes^23^ (Fig. 3D). Despite training on an identical dataset and task, the GNN classifier and AbRFC favor different AA substitutions, with the AbRFC selecting F,Y,D,E more frequently than any other model, while the GNN favors F, G, and P above their training set frequency. Notably, both RF models never predict G in the top 10% despite its prevalence in antibody paratopes as exhibited by the AbLang preference. We also noticed that the RF Regressor selected W mutations at a higher frequency than AbRFC, whereas AbRFC favored mutations to Y. To investigate, we examined the reported ΔΔ*G* for mutations of the form ${{{{{\rm{W}}}}}}\to {{{{{\rm{X}}}}}}$ and ${{{{{\rm{Y}}}}}}\to {{{{{\rm{X}}}}}}$ in the training set. We observed that mutations of the form ${{{{{\rm{W}}}}}}\to {{{{{\rm{X}}}}}}$ and ${{{{{\rm{Y}}}}}}\to {{{{{\rm{X}}}}}}$ were labeled non-deleterious at almost identical frequencies (0.186 vs 0.187) but using the mean ΔΔ*G* value favors ${{{{{\rm{W}}}}}}\to {{{{{\rm{X}}}}}}$ $(-1.67){{{{{\rm{over\; Y}}}}}}\to {{{{{\rm{X}}}}}}:(-1.37)$. Since there are >3 times as many examples of ${{{{{\rm{Y}}}}}}\to {{{{{\rm{X}}}}}}$ vs ${{{{{\rm{W}}}}}}\to {{{{{\rm{X}}}}}}$ in the dataset, it is natural that a classification model would predict a higher non-deleterious class probability for Y, whereas a model predicting ΔΔ*G* directly would give relatively more weight to W. This investigation provided further evidence that both model architecture and training task influence the mutations a model would suggest in practice.

### Performance on the OOD validation set

After fitting the AbRFC model on the entire training dataset, we assessed AbRFC’s performance on the OOD validation set. We used this dataset to accomplish two tasks. First, to understand whether AbRFC could effectively predict non-deleterious mutations and if so at which AbRFC score threshold. Second, to understand how AbRFC would perform in a practical setting by simulating in silico saturation mutagenesis. We then developed a protocol that incorporated the score threshold and rule-based ranking for selecting mutations.

**Fig. 4:**
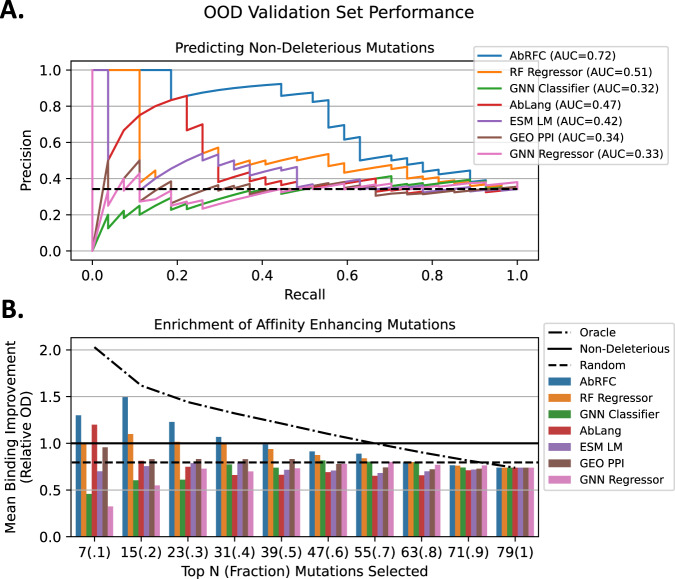
AbRFC predicts non-deleterious mutations in an OOD validation dataset. **A** Precision-Recall curves for AbRFC and 6 alternative models on the in-house OOD validation dataset. AbRFC, AbLang and the RF Regressor have significantly better-than-random performance on this dataset. **B** Enrichment for affinity enhancing residues was calculated by taking the mean binding improvement value associated with mutations ranked in the Top N by each model. AbRFC shows a robust ability to enrich for affinity enhancing mutations in the top (10, 20, 30, and 40%) of mutations selected.

Analysis of the OOD validation set shows that AbRFC delineates non-deleterious from deleterious mutations at a variety of AbRFC score threshold values. To define non-deleterious and deleterious point mutations using the previously collected ELISA-based screening assay data (Supplementary Table 1), we used a threshold cutoff of $\frac{O{D}_{{mutAA}}}{O{D}_{{refAA}}}\ge 1$ to consider a mutation non-deleterious. Figure 4A shows the PR curves for AbRFC and several published models, including the RF Regressor, the GNN Classifier, and 3 previously trained published methods (a GNN Regressor^10^, GeoPPI^24^, and the Efficient Evolution Language Models^7^) (Supplementary Methods). As in the validation set, the RF models outperform both the GNN classifier and AbLang. However, in this case AbLang outperforms the GNN Classifier. Interestingly, AbLang performs similarly on both tasks ($1.31* {AU}{C}_{{random}}$ on the training set and $1.38* {AU}{C}_{{random}}$ on the OOD validation set), likely because AbLang is not trained on any affinity data and thus the distribution of mutations sampled in the dataset is less of a factor. To understand the difference in performance between AbRFC and the RF Regressor, we examined which non-deleterious mutations were in the top 20 AbRFC ranked mutations but not in the top 20 RF Regressor mutations. 3/5 of these mutations (Supplementary Table 1) are on the periphery of the epitope, which explains why they are preferentially selected by a model predicting non-deleterious rather than affinity enhancing mutations.

We then examined whether AbRFC score ranking could identify affinity enhancing, rather than just non-deleterious, mutations. To this end, we ranked the validation set mutations by model score and calculated the average relative OD in the top-N mutations. AbRFC can identify affinity enhancing mutations in the top 10, 20, 30, and 40% of mutations (Fig. 4B). We used this information to select the AbRFC score threshold used to consider mutations during in silico saturation mutagenesis (0.60), which allows for the maximum sampling of mutations while maintaining average improvement over baseline.

To determine whether we could use this threshold alone to select mutations in practice, we simulated in silico saturation mutagenesis (Methods) using the PDB associated with the OOD validation set. This resulted in 1129 mutations across 65 positions, which we scored using AbRFC and resulted in 527 mutations (47%) with scores higher than 0.60. Since we intended to screen mutations on a single 96-well plate, we then analyzed the top 90 mutations ranked by model score. Immediately, we noticed that AbRFC oversampled specific positions and CDRs. For example, the top two most-sampled positions (L72 and L92) account for 21/90 = 23% of the total sampled mutations. Further, neither of these positions contains mutations in the interface as defined by ΔΔSASA. In fact, only 30 out of the top 90 mutations are in the interface, even though the frequency of such mutations in the dataset is 53%. Mutations were also unevenly distributed across CDRs (H1:7, H2:10, H3: 4, HFramework:9, L1:16, L2:5, L3:20, LFramework:19). Overall, the top 90 AbRFC-ranked mutations are biased towards non-interface and framework positions and the VL rather than the VH. Paratope-proximal non-interface residues are known to affect binding^25^, and so we did not want to eliminate such residues entirely. Rather, we wanted to control the frequency with which such mutations were sampled and to limit the oversampling of individual positions or CDRs.

We, therefore, established a process for selecting mutations to screen from the AbRFC ranked list. In addition to choosing only mutations above the AbRFC score threshold of 0.60 defined above, we limited the number of residues sampled at any given position to less than 10% of the total screen space. Finally, we ranked interface and non-interface residues separately so that at least 60% of screened residues were at interface positions. These three rules: AbRFC score ≥ 0.60, less than 10% of mutations at any one residue, and separate interface and non-interface rankings with a minimum of 60% of mutations in the interface were used to filter the AbRFC-ranked residues.

### Integrating AbRFC in a computational-experimental workflow

**Fig. 5:**
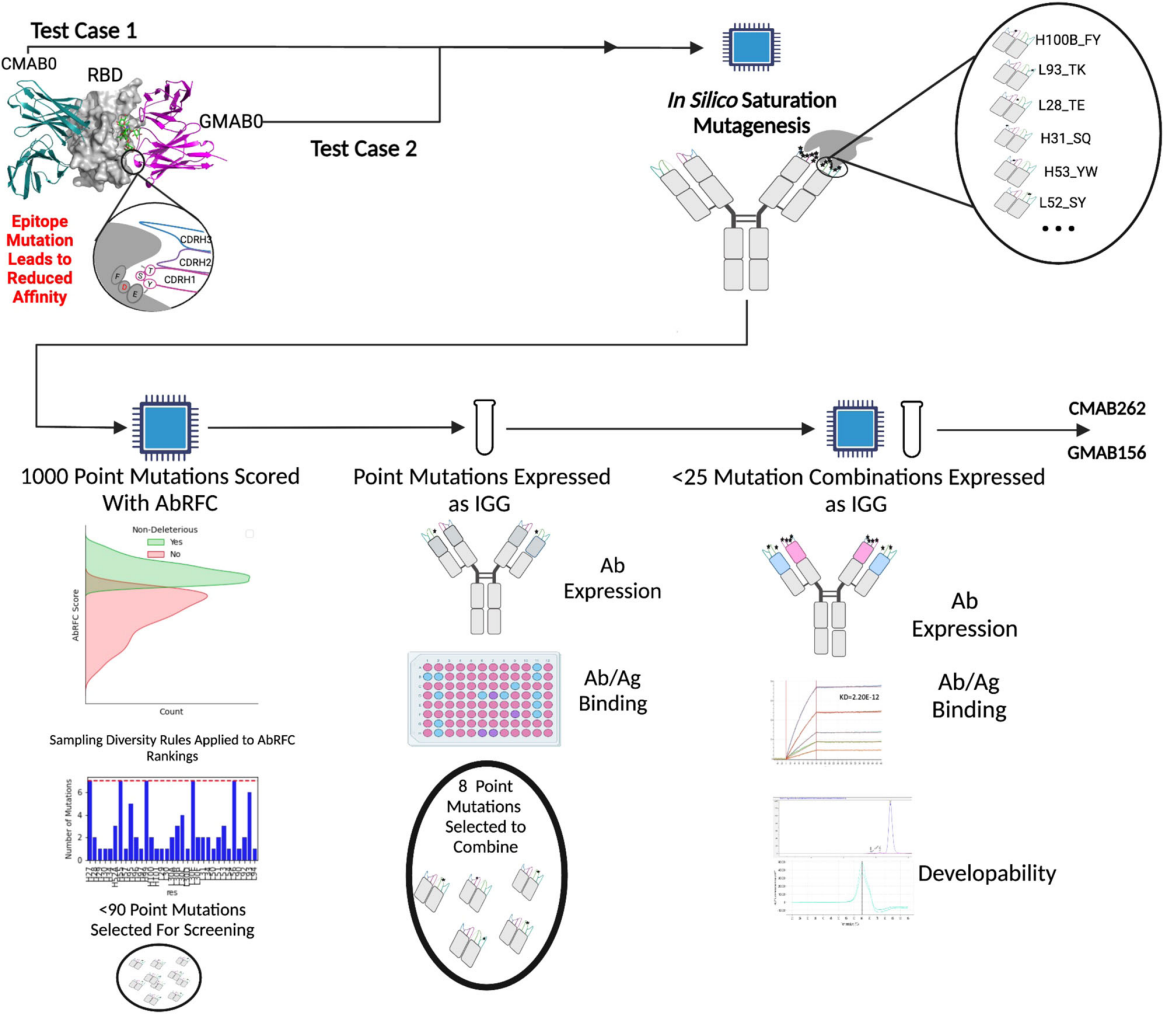
Integration of AbRFC into a computational-experimental platform. After selecting two template mAbs targeting unrelated epitopes on the SARS-COV-2 RBD with reduced affinity to the BA.1 strain, we modeled the BA.1 mutations on the available crystal structures of S309 and CR3022 with the B.1 RBD (Methods) and performed in silico saturation mutagenesis on the paratopes. These mutations were then ranked using AbRFC. Due to position-specific oversampling observed when we simulated the workflow on the OOD validation set, we applied pre-defined rules to limit the number of per-position residues sampled and ensure sufficient sampling of interface mutations (Results). These point mutations were then screened on full-length IGG constructs for expression and antigen binding using an ELISA-based assay. The affinity enhancing subset (≤8 mutations) were combined and screened by expressing (21,6) 2nd round constructs for the (CMAB,GMAB) programs respectively. To generate therapeutically viable candidates, developability-optimized FW regions were combined with the affinity enhanced CDRS, and the final constructs were screened using SEC and DSF in addition to Octet for antigen binding (see Methods).

Encouraged by the OOD performance of AbRFC on the validation set, we then set out to test AbRFC in a practical antibody engineering setting by integrating it into a computational-experimental workflow (Fig. 5). AbRFC was used to screen the mutations generated by in silico saturation mutagenesis so that the chosen mutations and controls could be expressed as full-length IGG and screened on a single 96-well plate. Mutations identified as affinity enhancing by this first round of screening were then combined in various permutations to generate final affinity-enhanced constructs that were screened in a second and final round of screening. This round included purity and melting temperature measurements in addition to expression and antibody binding.

To test AbRFC’s ability to rank mutations in different antibody-antigen systems, we chose two template mAbs targeting non-overlapping epitopes on the SARS-COV-2 Receptor Binding Domain (RBD). The criteria for selecting these mAbs were first that they targeted conserved epitopes on the RBD, second, that they had weakened affinity to the BA.1 strain, and third that they were dissimilar to each other and to any mAbs in the training set. Our analysis showed that a mAb targeting the CR3022^26^ epitope (called CMAB0 henceforth) and the S309 mAb^27^ (called GMAB0 henceforth) satisfied all these criteria (Supplementary Fig. 2). Importantly, we noted that the maximum VH identities of GMAB0 and CMAB0 with the training set were 0.74 and 0.59 respectively, ensuring that AbRFC had not seen similar examples during training (Supplementary Table 4). CMAB0 has matching Fab properties as that of CR3022 (Supplementary Fig. 3). With this information, we proceeded to apply our workflow to affinity enhance the template antibodies.

### AbRFC selects non-deleterious and affinity enhancing mutations

Saturation mutagenesis using the CMAB0 and GMAB0 complexes resulted in 954 and 597 mutations respectively (Methods). To compare performance across the systems, we selected experimental screen sizes of 75 (7.8%) and 50 (8.3%) mutations for CMAB0 and GMAB0 respectively. Applying the model threshold cutoff, we found that 40% (384) of CMAB0 and 38% (226) of GMAB0 mutations had ${AbRF}{C}_{{score}}\ge 0.6$. We limited the number of per-position mutations to less than 10% (7 for CMAB0, 5 for GMAB0) and ranked interface and non-interface residues separately, ensuring that >60% of mutations came from the interface (31 for GMAB0 and 50 for CMAB0) (Supplementary Data 1, Supplementary Data 2).

**Fig. 6:**
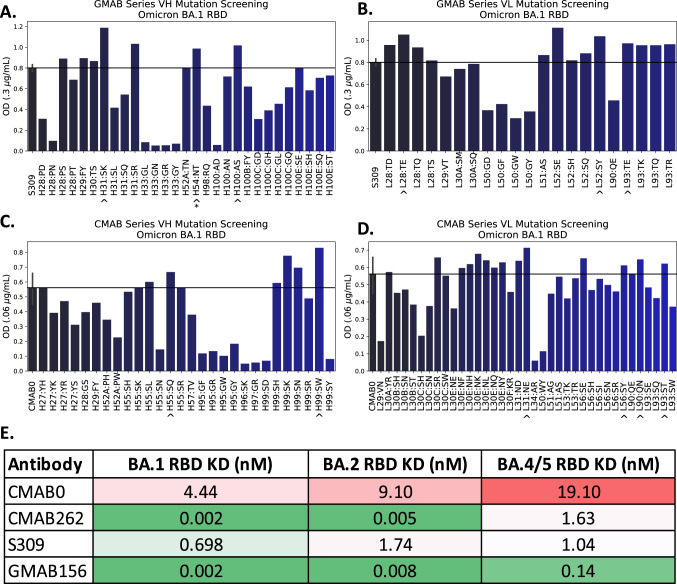
AbRFC identified several affinity enhancing mutations on both templates, resulting in 350–1000-fold affinity improvement when combined. **A**–**D** OD values in the linear range (at the concentration specified on the y-axis) for the GMAB VH, VL, and CMAB VH, VL point mutations, respectively. Point mutations denoted by ^ are the mutations present in the final candidate (CMAB262 or GMAB156, respectively). The H54:NT mutation is marked with * because it was selected manually to eliminate a known deamination site. **E** KD values by Octet BLI for the best-performing candidates generated by combining the top single mutations in the second round. The top 2nd round candidates, CMAB262 and GMAB156, show affinity improvements of 1480-fold and 350-fold, respectively, against the BA.1 RBD. These constructs also show affinity improvements against the BA.2 and BA.4/5 RBDs.

We then expressed the point mutations as full-length IGG1 antibodies and used an ELISA-based screening assay against the BA.1 RBD (Methods) to determine the relative binding of these mutations with respect to the templates (Fig. 6A, B, C, D). We observed that 14 out of the 64 (22%) experimentally tested CMAB0 mutations and 15 out of the 49 (31%) tested GMAB0 mutations exhibited improved binding relative to the respective template ($\frac{{OD}(A{A}_{{mut}})}{{OD}(A{A}_{{template}})}\ge 1.1$), validating the filtering hypothesis that predicting non-deleterious mutations is sufficient to uncover affinity enhancing mutations.

To determine if the AbRFC score threshold of 0.60 discriminated beyond the top-ranking mutations, we screened ten additional mutations on CMAB0 and eight additional mutations on GMAB0 at a range of AbRFC scores [0.15–0.75] that were manually selected using structural information and in silico scores (Supplementary Data 1, Supplementary Data 2). On CMAB0 3/6 mutations in the predicted non-deleterious subset showed improved binding, and 0/4 mutations with AbRFC scores below the 0.60 threshold showed binding. For the GMAB0 subset, none of the mutations with AbRFC score ≥0.6 (0/6) or <0.6 (0/2) subset showed improved binding. This hit rate for identifying mutations with improved affinity (25%) is very similar to the hit rate of the AbRFC ranked mutations (26%), providing more evidence for the filtering hypothesis, and revealing that there are affinity enhancing mutations outside of the Top-N ranking by AbRFC score.

### Combining promising mutations yields 350 and > 1000 fold affinity improvement against BA.1

Given the higher OD values of the single point mutations above, we reasoned that combining several of these mutations would significantly enhance the affinity of the constructs. Notably, unlike in previous approaches where mutations were combined and screened in a stepwise fashion over multiple rounds (e.g., two mutations, three mutations, …)^10^, we posited that combining several affinity enhancing mutations at once would provide the additive effect required (Methods). This is analogous to recent work showing the high accuracy of single point mutations in predicting combination effects in other use cases^28^.

Analysis by Octet BLI (Methods) showed that the best 2nd round candidate on the CMAB track (CMAB262) had an affinity increase of >1000-fold over CMAB0 against BA.1 (Fig. 6E, Supplementary Fig. 4). The mutations on this candidate included the two top performing VH single point mutations 55_SQ and 99_SW from round 1, as well as the 4 VL mutations (31_NE, 90_QN, 56_SY, 93_ST). Notably, the residues H99, L90, and L93 are all within 3.8 A of each other in the CMAB0 structural model, showing that combining proximal residues with affinity-enhancing effects may have an additive effect.

The top performing 2nd round candidate on the GMAB track (GMAB156) exhibited 317-fold affinity improvement relative to GMAB0 (Fig. 6E). In this track the first round screening suggested 3 spatially distinct mutations per chain (H31_SK, H54_NT, H100_AS and L28_TE/Q, L52S_E/Y, and L93T_E/K), with the VL of GMAB156 containing 28_E, 52_Y, and 93_E. Only the non-interface mutation H54_NT, selected to eliminate a known deamination site, was not from the ranking procedure. The VL mutations suggest that multiple non-deleterious or even affinity enhancing residues are possible at a given residue.

To ascertain the extent to which these mAbs could maintain binding superiority across multiple variants, binding to BA.2 and BA.4/5 was also tested. Both candidates maintained binding affinity advantages against these variants, with 3000-fold for BA.2 and 32-fold for BA.4/5 for CMAB262 and 230-fold and 7.5-fold for GMAB156 (Fig. 6E). This data shows that mAbs engineered against one strain may carry their properties to other strains, but that strain-specific mutations can affect the relative affinity advantage.

## Discussion

In silico antibody design is a rapidly evolving field that leverages the power of computational methods and molecular modeling to create epitope-specific antibodies with specific properties. While de novo design of epitope-specific antibodies is still a challenge, optimization of antibodies targeting specific epitopes using structure-guided metrices is feasible, as demonstrated by previous studies^12,29,30^. The integration of ML has ignited significant interest in the field of antibody optimization. We demonstrate herein, with two examples, that despite the limitations of the publicly available training data, a tree-based classification model using structure-guided metrics as features can successfully prioritize mutations for affinity enhancement.

One of the earliest efforts to introduce ML in computational antibody engineering was conducted by our group^12^ where we combined feature engineering and logistic regression to develop a predictive model for discriminating native antigen-antibody poses from decoys. Classical ML models can work on smaller (training) datasets and are computationally cheap and readily interpretable. In contrast to these models, DL techniques require extremely large datasets to train and are difficult to interpret owing to their “black box” nature. While tools like mCSM-AB2^31^ have employed classical ML to determine the impact of mutations on antibody binding, this represents the first study (to the best of our knowledge) to show that antibodies having improved properties (e.g., affinity) can be generated within a small experimental screen space using classical ML.

The classical ML platform presented here is distinct in the following aspects. First, we reduce the challenge of predicting the most affinity enhancing mutations to a classification problem, where we train a model to discriminate deleterious mutations from neutral or affinity enhancing mutations in the paratope. Consequently, ranking mutations by AbRFC and imposing limits on residue-specific oversampling resulted in a set of mutations that contained enough affinity enhancing mutations for achieving >300-fold affinity improvement in 2 unrelated systems. Second, we base our approach on examining single mutations to develop antibodies with increased affinity, predicated on the hypothesis that these mutations will exert a cumulative effect when combined. Recent work on activity cliffs in small molecules^32^, an analogous situation where very small differences can have large impact, supports the idea of the continued use of classical ML for the problems involving predicting impact of small molecular changes on overall function and other properties, despite the exciting capacity of DL to generate large sets of highly diverse sequences.

While building AbRFC, we noted that our choice of featurization and the training dataset led to certain biases, including the failure to sample mutations to Glycine during cross-validation. However, Glycines can contribute to the interface, directly or indirectly, leading to improved antigen-antibody affinity^33^. Knowing this limitation of AbRFC, we experimentally sampled Glycine mutations predicted by two other methods, AbLang and ΔΔ*G*, resulting in a construct (CMAB283) that showed approximately the same binding improvement as CMAB262 on BA.1 and BA.2, maintained the >1000 fold improvement on BA.4/5 and also showed synergistic potent in vitro neutralization of BA.1 and BA.2 pseudotyped viruses when combined with GMAB156 (Supplementary Table 5, Supplementary Fig. 5). This exercise further underscored the limitations of the training data, emphasizing the need to enrich it with a diverse and high-quality set of mutations for the creation of more effective models.

We were required to add rule-based selection criteria for filtering the AbRFC rankings, given that we used in silico saturation mutagenesis to generate mutations. However, it is possible to envision in the future that using generative modeling to sample from the distribution of somatic hypermutations associated with a template clonotype and subsequently scoring them with the platform described here would achieve a fully end-to-end AI system. Such an end-to-end AI system would eliminate the need to perform in silico saturation mutagenesis and rule-based filtering. Further, the model described here can be developed further to predict and score candidates that have multiple mutations, decreasing the experimental iteration time still further.

In summary, the Random Forest classifier, AbRFC, described in this study is a valuable tool for affinity enhancement given a template antibody and its structural interaction with the antigen or its variants. Importantly our platform described herein can be readily integrated into a computational-experimental cycle to achieve affinity enhancement using only two rounds of experimentation.

## Methods

### Training dataset

The training dataset was downloaded from (https://biosig.lab.uq.edu.au/mcsm_ab2/data). This dataset contains 905 single-point mutation datapoints from the SKEMPIV2 database representing 49 Ab-Ag structures, three nanobody structures, and eight general protein-protein interaction structures. This dataset also contains 905 augmented datapoints where the mutation order of the initial observations is reversed and the ΔΔ*G* is multiplied by −1. We used the training dataset as downloaded, with the exception of removing ten observations where the reference or mutant AA was a Cys.

### Cross-validation splits

Five-fold CV splits were generated using the “Complex ID” column present in the training dataset, ensuring that all mutations from a given Complex ID were grouped into either the training or validation set. To further reduce data leakage, we analyzed whether there could be data leakage due to mutations from “homologous complexes.” We defined homologous complexes $({comple}{x}_{1} \sim {comple}{x}_{2})$ separately for Ab-Ag, Nanobody, and non-IG PPIs in the dataset. For Ab-Ag, 2 complexes were considered homologous if the heavy variable region sequence identity (VH ID) and light variable region sequence identity (VL ID) were both ≥0.90. Additionally, we manually checked all PDBs where the VH ID or VL ID was ≥0.90 to ensure similar antibodies targeting the same epitope were classified as homologous. There are also three nanobody PDBs in the dataset (4KRO, 4KRL, 4KRP) two of which target the same epitope (4KRO and 4KRP)—we considered these two complexes homologous. Finally, there are eight non-IG PPI complexes in the dataset, two of these (1FCC and 1FC2) have chains that share 100% sequence identity and were classified as homologous while the other six share <10% sequence identity. Supplementary Data 3 contains the pairwise PDB sequence identities for all PDBs in this dataset.

To avoid data leakage due to these homologous complexes, we ensured that no position-specific information was shared between homologous complexes in the training and validation subsets. That is, for each fold given a mutation ${M}_{v}=({comple}{x}_{v},{chai}{n}_{v},{positio}{n}_{v},{muta}{a}_{v})$ in the validation subset, we removed any training subset mutation ${M}_{t}=({comple}{x}_{t},{chai}{n}_{t},{positio}{n}_{t},{muta}{a}_{t})$ when $({comple}{x}_{v} \sim {comple}{x}_{t})$ and (${chai}{n}_{v}={chai}{n}_{t}$) and $({positio}{n}_{v}={positio}{n}_{t})$.

### In silico saturation mutagenesis

Given a structure of the template PDB in complex with the antigen, residue positions to be mutated were selected by considering any position that had a heavy atom within 10 A of the antigen in the starting structure. To avoid disrupting highly conserved residues in the antibody variable region, the following positions were ignored: template AA is Cysteine; template AA is Glycine AND is 90% conserved in human antibody sequences; template AA is any amino acid AND 95% conserved in human antibody sequences. All AAs except Cysteine and AAs that would introduce glycosylation were considered. These mutations were introduced into the structure and features were generated from the structures as detailed below.

### Feature extraction from structure

All structures, regardless of the dataset, were processed identically. PDBs associated with the relevant complexes were downloaded from the RCSB Protein Data Bank. The training dataset file available from the link above contains the names of all PDBs used for training. For the validation dataset, we used PDB 5WHK. For CMAB0, we used the PDBs 6YLA and 7LOP. For GMAB0 (S309), we used the PDBs 7R6W and 7BEP.

Structures were cleaned by renaming heavy and light chains to (H, L respectively) and renumbering them using the Chothia numbering scheme. Structures associated with the original (WT) complex were relaxed ten times using Rosetta Fast Relax with the identical parameters to those used in the RosettaAntibodyDesign protocol. The lowest energy structure was selected and used for feature extraction. For each mutation, a new PDB file was generated using PyRosetta, wherein side chains within 5 A heavy atom distance of the mutation were repacked. A new file was also generated for the reference AA with repacking at 5 A to there were no artifacts from the repacking procedure. These two files (${PD}{B}_{{ref}}$ and ${PD}{B}_{{mut}}$ respectively) were used to generate the features.

Features were extracted from structure using the following procedure. Given the repacked reference ${PD}{B}_{{ref}}$ and mutant ${PD}{B}_{{mut}}$ PDBs, all features $f$ were calculated by first calculating the features $f({PD}{B}_{{ref}})$ and $f({PD}{B}_{{mut}})$ and subtracting such that $f=f({PD}{B}_{{ref}})-f({PD}{B}_{{mut}}).$ Features were generated for the mutated residue, the 1st order neighbors of the mutated residue, the 2nd order neighbors of the mutated residue, the interface, and the entire complex. Features are annotated using a _0 if they apply to the mutated residue itself, a _1 if they apply to the 1st order neighbors and a _2 if they apply to the 2nd order neighbors. Notably no 2nd order neighbor features were selected during feature selection. To define 1st and 2nd order neighbors, we used the neighborhood graph defined by adding an edge between any two residues where the maximum absolute Rosetta Energy term (among “fa_atr”, “fa_rep”, “fa_sol”, “fa_intra_rep”, “fa_intra_sol_xover4”, “lk_ball_wtd”, “fa_elec”, “pro_close”, “hbond_sr_bb”, “hbond_lr_bb”, “hbond_bb_sc”, “hbond_sc”) was greater than 0.05 (0.15 for 2nd degree). In addition to these local features, interface features (calculated using the InterfaceAnalyzerMover in Pyrosetta) and full complex features (e.g., total Rosetta energy change and the SIN normalization constant) were used. Supplementary Table 2 contains a list of all the features engineered to be tested in AbRFC.

### **ΔΔ*G*** Cutoff Selection

To determine a **ΔΔ*G*** cutoff, we examined the tradeoff between having a large class imbalance and defining a cutoff that would be sensitive to experimental noise. The established $|{{{{{\boldsymbol{\Delta }}}}}}{{{{{\boldsymbol{\Delta }}}}}}{{{{{\boldsymbol{G}}}}}}|$< 0.4 for determining non-hotspots translates to a fold change $|\frac{K{D}_{{wt}}}{K{D}_{{mut}}}|\le 2$, which is a reasonable definition to avoid classifying mutations as hotspots based on experimental noise. Because the training dataset has been augmented to be symmetric, a $\Delta \Delta G\ge -0.4$ cutoff means that 67% of the examples are in the non-deleterious class, including both mutations in any pair where the experimentally determined mutation is |$\Delta \Delta {G|}\le 0.4$, which includes 34% of the dataset. To avoid this imbalance, we wanted to select a $\Delta \Delta G$ above -0.4 but less than 0 so that mutations within experimental noise were non-deleterious. To establish experimental noise, we used the full SKEMPIV2 dataset to estimate how much fold-change typically varies. We took all mutations where there was more than one experimental measurement for the same mutation (PDB, chain, mutant AA) and found that, after removing outliers $({FC} > 10)$ the ${mean}\left({STD}\left({FC}\right)\right)=0.28$. Therefore, we selected a $\frac{K{D}_{{wt}}}{K{D}_{{mut}}}\ge 0.7$ ($\Delta \Delta G\ge -0.21$), which also reduces the number of mutation pairs where both the experimentally determined and augmented mutation are in the same class to 23%. To train AbRFC we used $${y}_{{train}}=\left\{\begin{array}{c}0, \Delta \Delta {{{{{\rm{G}}}}}} \, < -0.21\\ 1, \Delta \Delta \, {{{{{\rm{G}}}}}}\ge 0.21\end{array}\right.$$ to assign mutations to the non-deleterious (${y}_{{train}}=1$) or deleterious class.

### AbRFC model implementation

The AbRFC model was implemented using the “RandomForestClassifier” class available in the python package scikit-learn (sklearn) (https://scikit-learn.org/stable/modules/generated/sklearn.ensemble.RandomForestClassifier.html). After features were extracted from the PDBs as described above, we built a sklearn “Pipeline” (https://scikit-learn.org/stable/modules/generated/sklearn.pipeline.Pipeline.html) consisting of an “IterativeImputer“ (https://scikit-learn.org/stable/modules/generated/sklearn.impute.IterativeImputer.html), a “StandardScaler” (https://scikit-learn.org/stable/modules/generated/sklearn.preprocessing.StandardScaler.html) and a “RandomForestClassifier”. The “IterativeImputer” was required because AIF scores cannot be computed for the non-Ab/Ag complexes in the training set and needed to be imputed. The “StandardScaler” transforms all numerical features into their standard scores. This “Pipeline” was applied to the extracted features to score mutations.

The “AbRFC score” of a mutation resulted from applying the “predict_proba“ method of the “RandomForestClassifer” to the features associated with that mutation. Briefly, the “predict_proba“ method estimates the probability of a mutation belonging to the non-deleterious class by averaging the probability prediction from each tree as described in the documentation. We refer to the output as the “AbRFC score” rather than as a probability because it is known that such estimates are not always well-calibrated, meaning they do not reliably approximate the true class probability, especially on OOD data.

Model optimization was performed by tuning several hyper parameters available in the “RandomForestClassifier“ class using grid search during CV. PR AUC on the validation subset was the metric to be optimized. These included criterion [“gini”,“entropy”], max_depth,and min_samples_leaf. Although this grid search was extensive, we found that max_depth and min_samples_leaf performed similar functions and therefore, the main differences in model performance and regularization occurred from tuning min_samples_leaf as described in the Results section. The n_estimators parameter, which determines the number of trees in the forest, was set to 1000 to further reduce model variance.

### Structural modeling and scoring of GMAB0 and CMAB0 mutations

All available structures at the time of this work for GMAB0 (7BEP, 7R6W) and CR3022 (6YLA,7LOP) had the mAbs in complex with the B.1 RBD. Because BA.1 was the strain of interest, we introduced the BA.1 mutations within 10 A of either antibody (S371L,S373P,S375F,N440K,G446S) onto the structures using PyRosetta and repacked side chains within 5 A. In addition, we modeled CMAB0 by porting the AA differences between CMAB0 and CR3022 onto both PDBs.

Saturation mutagenesis was performed as described above. GMAB0 targets a glycoepitope, meaning that a portion of the antibody is in contact with the RBD glycan at position N343. Ab-glycan interactions have very limited high-quality training and so were not considered during the featurization for AbRFC. Therefore, when performing saturation mutagenesis for GMAB0 we avoided sampling residues within 4 A of the N343 glycan in 7BEP.

Two PDBs were used for each template mAb to mitigate structural variability. After features were extracted from the two PDBs as described above, scores from AbRFC using both sets of features were averaged, and this average score (“AbRFC score” column in Supplementary Data 1 and Supplementary Data 2) was used to rank mutations as described in the results.

### Combining mutations for second round designs

Individual point mutations were selected in the computational-experimental workflow based on relative binding in the ELISA-based screening assay (see Results section). To optimize the developability of the candidates, CDR loops carrying sets of these mutations were combined with several different antibody framework regions. Frameworks were selected using an identical procedure to that described in ref. ^16^. Briefly, filters for the GMAB0-based designs included:

H1 north cluster = H1-13-A, H2 north cluster = H2-10-A, H3 length = 18, maximum number of PTMS: 18, maximum rare amino acids: 1, minimum V/J-germline ID ≥ 0.8.

L1 north cluster = L1-12-B, L2 north cluster = L2-8-A, L3 north cluster = L3-8-A, maximum number of PTMs = 6, maximum rare amino acids = 2, minimum V/J-germline ID ≥ 0.81. Filters for the CMAB0-based designs included: H1 north cluster = H1-13-A, H2 north cluster = H2-10-A, H3 length = 10, maximum number of PTMS: 12, maximum rare amino acids: 0, minimum V/J-germline ID ≥ 0.86.

L1 north cluster = L1-16,17-A, L2 north cluster = L2-8-A, L3 north cluster = L3-9,10-A, maximum number of PTMS = 7, maximum rare amino acids = 0, V/J-germline ID ≥ 0.80.

Scaffold-CDR combinations were ranked according to Rosetta Energy, the developability metrics from Therapeutic Antibody Profiling, and sampled to maximize framework diversity as described previously.

### Expression and purification of recombinant monoclonal antibodies

The variable heavy and light chain sequence of anti-SARS CoV2 antibodies S309, and CMAB0 16 and variants were cloned into the full-length IgG1 expression vectors pcDNA3.3 HC and pcDNA3.3 LC (ATUM). The recombinant antibodies were transiently expressed in both ExpiCHO and Expi293 cells according to manufacturer’s protocol (Gibco). The clarified cell culture supernatants from 1 mL transient transfections of the antibodies were affinity purified using the AssayMAP BRAVO platform with AssayMAP 5 µL Protein A (PA-W) cartridges (Agilent Technologies, Cat#5496-60000). Recombinantly expressed antibodies from larger scale transient transfections were affinity purified from clarified cell culture supernatants on the ӒKTA pure™ chromatography system using MabSelect PrismA™ protein A chromatography resin (Cytiva). The purified recombinant monoclonal antibodies were stored in 1X phosphate buffered saline, pH7 0.4 (Gibco, Cat#10010023), at 4 °C until use. Specific site directed mutations on the S309 and CMAB0 antibody sequence was done using the QuikChange II site directed mutagenesis kit (Agilent Technologies, Cat#200522).

### Screening of expressed recombinant antibodies using enzyme linked immunosorbent assay (ELISA)

The antibodies purified from a 1 mL transient transfection was tested for binding against BA.1 RBD (Acro# SPD C522e) protein on an ELISA. Briefly, 2 μg/mL of SARS CoV2 BA.1 RBD protein were coated on 96-well ELISA plates (Nunc Maxisorp) and left overnight at 4 °C. The wells were blocked with 5% Blotto (Santa Cruz) in 1xPBST for 1 h at room temperature. Using the Opentrons OT-2 benchtop liquid handler, the purified variant recombinant antibodies based on S309 and CMAB0 were diluted to either 12 and 0.3 μg/mL or 12 and 0.06 μg/mL respectively and added to the plates and incubated on a rocker platform for 2 h at room temperature. After rinsing the plates three times with 1xPBST a rabbit anti-human IgG conjugated to horseradish peroxidase (Jackson Immuno Research) was added to each well. The plates were incubated for 1 h at room temperature followed by washing with 1xPBST and addition of TMB substrate. The reaction was stopped by adding 1 N sulfuric acid and the absorbance was read at 405 nm.

Select CMAB and GMAB candidates were serially diluted and tested for binding against BA.1 RBD (Acro# SPD C522e) protein on an ELISA to determine apparent KD values. Briefly, 0.5 μg/mL of BA.1 RBD protein were coated on 96-well ELISA plates (Nunc Maxisorp) and left overnight at 4 °C. The wells were blocked with 5% Blotto (Santa Cruz) in 1xPBST for 1 h at room temperature. Using the Opentrons OT-2 benchtop liquid handler, a three-fold serial dilution of select antibodies from 9 μg/mL to 0.152 ng/μL was made and added to the plates and incubated on a rocker platform for 2 h at room temperature. After rinsing the plates three times with 1xPBST a rabbit anti-human IgG conjugated to horseradish peroxidase (Jackson Immuno Research) was added to each well. The plates were incubated for 1 h at room temperature followed by washing with 1xPBST and addition of TMB substrate. The reaction was stopped by adding 1 N sulfuric acid and the absorbance was read at 405 nm.

### Affinity determination using octet (biolayer interferometry)

The affinity of recombinantly expressed CR3022 and CMAB0 to SARS-CoV-2 RBD (Acro# SPD-C52H3) was determined using AHC sensors. The RBD was diluted twofold fold starting at 125 nM in 1x Kinetics buffer. The affinity of select recombinantly expressed antibodies to BA.1 RBD (Acro# SPD C522e), BA.2 RBD (Acro# SPD-C522g), and BA.4/5 RBD (Acro# SPD-C522r), was determined using Pro A sensors. A twofold dilution of the different RBDs from 60 nM to 1.875 nM in 20 mM HEPES with 150 mM NaCl and 0.05% tween 20, pH 7.4 was made. The antibody coated Pro A/AHC sensors were then associated in the various dilutions followed by dissociation in the same buffer. The KD values were calculated using the global fit method on the Octet Red96 (Sartorius) instrument.

### Reporting summary

Further information on research design is available in the Nature Portfolio Reporting Summary linked to this article.

### Supplementary information

Supplementary Material

Description of Additional Supplementary Files

Supplementary Data 1

Supplementary Data 2

Supplementary Data 3

Reporting Summary

## Data Availability

The original data contributions of this article are included in the article/Supplementary Information. The publicly available data used in this study are available as follows. The collated dataset of experimental data used to train and evaluate AbRFC and other models is available from the mcsm-Ab2 website (https://biosig.lab.uq.edu.au/mcsm_ab2/data). All PDBs for this dataset, the validation dataset, and for the 2 SARS-COV-2 test examples were downloaded from the Protein Data Bank (https://rcsb.org). Large datasets support the analysis and conclusions are supplied as individual files, Supplementary Data 1, Supplementary Data 2 and Supplementary Data 3. The source data supporting Fig. 6 has been uploaded to https://github.com/tbc01/AbRFC.

## Code availability

The code and relevant data to reproduce the results in this study is available at https://github.com/tbc01/AbRFC. Further inquiries can be directed to the corresponding authors.
