# Supplementary material for: Enhancing antibody affinity through experimental sampling of non-deleterious CDR mutations predicted by machine learning
Source: Commun Chem. 2023 Nov 9;6:244. doi: 10.1038/s42004-023-01037-7 (PMC10636138; doi:10.1038/s42004-023-01037-7)
Supplement: Supplementary file 1 — Supplementary Material [file 42004_2023_1037_MOESM1_ESM.pdf]

Supporting Online Information for

Enhancing Antibody Affinity Through Experimental Sampling of Non-Deleterious CDR Mutations Predicted by Machine Learning

Supplementary Methods

Random Forest Regressor Implementation: The Random Forest Regressor was implemented using identical parameters to the Random Forest Classifier (**Methods**), except for those parameters that are classification specific (min_impurity_decrease, class_weight, criterion). The default loss function "squared error," was used.

GNN Model Classifier Implementation: The code for the graph neural network implemented in Shan S et. al., [1] was cloned from https://github.com/HeliXonProtein/binding-ddg-predictor. To retrain the network as a classifier rather than a regressor, a sigmoid layer was added to the final layer readout to map the embeddings to [0,1]. We also updated the loss function from the mean squared error loss to the cross entropy loss, accommodating the classification problem. Very specifically, the original network returns the variable `per_residue_ddg.sum(axis=1)`. We simply wrapped this in a sigmoid function `torch.sigmoid(per_residue_ddg.sum(axis=1))`. The cross entropy loss was implemented using `torch.nn.crossentropyloss()`.

AbLang Model Implementation*:* The AbLang [2] package was installed via PyPi. The pre-trained models for the heavy and light chains were downloaded. The likelihoods for mutations at each position were scored using the `model(seqs,mode='likelihood') command, and the logits were converted into probabilities using the softmax function, and the AbLang score used was the probability ratio $\frac{P_{AbLang}(mut_{AA})}{P_{AbLang}(ref_{AA})}$.

GNN Model Regressor Execution: The model was downloaded from https://github.com/HeliXonProtein/binding-ddg-predictor and the `predict.py` script run as instructed in the README.md documentation. The same structures scored by AbRFC were input as the wild type and mutant pdbs for the predict.py script.

GEOPPI Model Execution: GEOPPI [3] was cloned from https://github.com/Liuxg16/GeoPPI.git` on 09/27/2021. Mutation candidates were evaluated using the command `python run.py [pdb file] [Mutation] [partnerA_partnerB]` as described in the README.md file.

ESM Language Model Execution: The code from [4] was downloaded from the corresponding github repository (https://github.com/brianhie/efficient-evolution). The same ESM models were used to score the validation set mutations. Because the process in [4] selects specific amino acids rather than scoring each mutation, we instead extracted a score for each mutation by computing $\mathrm{score}_{\mathrm{model}} = \frac{P_{model}(Mut)}{P_{model}(WT)}$.

Pseudovirus neutralization assay: For the pseudovirus neutralization assay, HEK 293T cells expressing ACE2 and TMPRSS2 were purchased from Genecopoeia (Catalog # SL222). The pseudovirus particles used in the study were procured from eEnzyme (catalog #s SCV2-PsV-Omicron, and SCV2-PsV-OmiBA2). The HEK 293T cells were maintained in DMEM (Corning Catalog# 10-013-CV) containing 10% FBS (Gibco Catalog # A38401-01) containing selection

antibiotics hygromycin and puromycin as per the manufacturer's protocol. The test antibodies were incubated with the pseudovirus particles for 1h at 37°C. Afterwards, HEK 293T cells expressing hACE2 and TMPRSS2 were incubated with the antibody/pseudovirus mixture and incubated for 48h. After 48hours incubation, the luciferase activity of SARS-CoV-2 pseudovirus infected HEK 293T cells were determined by luciferase reporter assay kit (eEnzyme, Catalog # CA-L165-10). e. The relative luciferase activity (%) was calculated as follows:

$$\left[\frac{mean\ RLU\ antibody\ treated\ sample - mean\ RLU\ from\ cell\ only\ control}{mean\ RLU\ for\ virus\ only\ sample - mean\ RLU\ from\ cell\ only\ control}\right] * 100$$

Results of neutralization assays were plotted by normalization to samples where no antibody was used, and the half-maximal inhibitory concentration (IC50) was calculated using 4-parameter non-linear regression using GraphPad Prism. Each experiment was run in duplicate.

Supplementary Tables

Table S1. Out of Distribution Validation Set. Mutations (Chothia Numbering) and relative ODs for the validation dataset consisting of expert selected mutations for a previous affinity enhancement campaign. The 5 bolded mutations are mutations ranked in the top 20 of the Antibody Random Forest Classifier (AbRFC) but not by the Random Forest Regressor. The underlined mutations are on the periphery of the paratope.

Mutation	ELISA Relative OD (1:10000 Dilution)	Mutation	ELISA Relative OD (1:10000 Dilution)
H30_SK	1.00	<u>**L27_GS**</u>	1.12
H30_SN	0.96	L30_VD	0.76
H30_SR	1.05	L30_VN	0.54
H31_ET	1.08	L30_VS	0.94
H33_AV	0.05	L30A_GD	0.43
H33_AY	0.10	L30A_GE	0.44
H50_SI	0.08	L30A_GM	0.37
H50_SV	0.13	L30A_GN	0.80
H52_GI	0.06	L30B_SK	0.62
H52_GK	0.05	L30B_SQ	0.64
H52_GV	0.05	L30C_YF	1.41
H52A_SA	0.86	L30C_YI	1.01
H52A_ST	0.28	L30C_YK	0.17
H52A_SY	0.07	L30C_YR	0.31
H53_SK	0.36	L31_NS	1.22
H53_SN	0.34	L32_LF	0.50
H53_SR	0.77	L32_LY	0.25
H53_ST	1.01	L51_DE	0.53
H54_GN	0.25	L53_QA	1.21
H55_GN	0.88	L66_KR	0.74
H55_GS	1.16	L66_KS	0.96
H55_GT	1.16	<u>**L67_SD**</u>	1.28
H56_QI	1.08	<u>**L67_SE**</u>	1.28
H56_QK	1.31	L71_AV	0.82
H56_QN	0.62	L89_AS	2.26
**H56_QR**	1.11	L91_YK	0.20
H56_QV	1.12	L91_YR	0.43
H58_KI	0.47	L92_AK	1.95
H58_KR	1.17	L92_AQ	1.69
H58_KV	0.83	L92_AR	2.53
H94_RK	0.20	L93_GS	0.17
H94_RT	0.09	L94_SK	1.94
H94_RY	0.08	L94_SQ	1.56
H96_AF	0.26	L94_SR	2.26

H96_AL	0.11	L94_SV	0.76
H97_IF	0.41	L95_GI	0.13
H97_IS	0.10	L95_GV	0.18
H97_IW	0.41	L96_YF	0.54
H97_IY	0.06		
**H98_GA**	1.19		
H98_GV	1.03		

Table S2. **Features Considered for AbRFC**. These are the details of the features, the grouping they belong to, and whether they were used in the final model (value of 1 if used and 0 if not used). For the descriptions that refer to the "Rosetta Score Function" [5] please refer to https://new.rosettacommons.org/demos/latest/tutorials/scoring/scoring. Additionally, the features that have a prefix of "i" were all calculated using the InterfaceAnalyzerMover class from Pyrosetta [6], which is documented here: https://graylab.jhu.edu/PyRosetta.documentation/pyrosetta.rosetta.protocols.analysis.html#pyrosetta.rosetta.protocols.analysis.InterfaceAnalyzerMover.

Feature	Description	Feature Group	In Final Model
aif_score	Amino acid Interface Fitness	Mutated Residue	1
dE2	Total Rosetta Energy Score	Full Complex	1
fa_atr_0	See Rosetta Score Function	Mutated Residue	1
fa_atr_1	See Rosetta Score Function	1st Degree Neighbors	1
fa_elec_0	See Rosetta Score Function	1st Degree Neighbors	1
fa_elec_1	See Rosetta Score Function	1st Degree Neighbors	1
fa_intra_rep_0	See Rosetta Score Function	1st Degree Neighbors	0
fa_intra_rep_1	See Rosetta Score Function	1st Degree Neighbors	0
fa_intra_sol_xover4_0	See Rosetta Score Function	1st Degree Neighbors	0
fa_intra_sol_xover4_1	See Rosetta Score Function	1st Degree Neighbors	0
fa_rep_0	See Rosetta Score Function	1st Degree Neighbors	1
fa_rep_1	See Rosetta Score Function	1st Degree Neighbors	1
fa_sol_0	See Rosetta Score Function	1st Degree Neighbors	1
fa_sol_1	See Rosetta Score Function	1st Degree Neighbors	1
hbond_bb_sc_0	See Rosetta Score Function	1st Degree Neighbors	1
hbond_bb_sc_1	See Rosetta Score Function	1st Degree Neighbors	1
hbond_lr_bb_0	See Rosetta Score Function	Mutated Residue	0
hbond_lr_bb_1	See Rosetta Score Function	1st Degree Neighbors	0
hbond_sc_0	See Rosetta Score Function	Mutated Residue	1
hbond_sc_1	See Rosetta Score Function	1st Degree Neighbors	0
hbond_sr_bb_0	See Rosetta Score Function	Mutated Residue	0
hbond_sr_bb_1	See Rosetta Score Function	1st Degree Neighbors	0

icomplexed_sasa_0	Solvent Accessible Surface Area when complexed	Mutated Residue	0
icomplexed_sasa_1	SASA when complexed	1st Degree Neighbors	0
icomplexed_sasa_2	SASA when complexed	2nd Degree Neighbors	0
idelta_unsat_hbonds	Change in number of unsatisfied hydrogen bonds on complexation	Interface	1
idG_0	$\Delta\Delta G$	Mutated Residue	0
idG_1	$\Delta\Delta G$	1st Degree Neighbors	1
idG_2	$\Delta\Delta G$	2nd Degree Neighbors	0
idhSASA_0	Hydrophobic SASA	Mutated Residue	0
idhSASA_1	Hydrophobic SASA	1st Degree Neighbors	1
idhSASA_2	Hydrophobic SASA	2nd Degree Neighbors	0
idhSASA_rel_by_charge_0	Relative Hydrophobic SASA	Mutated Residue	0
idhSASA_rel_by_charge_1	Relative Hydrophobic SASA	1st Degree Neighbors	0
idhSASA_rel_by_charge_2	Relative Hydrophobic SASA	2nd Degree Neighbors	0
idhSASA_sc_0	Side Chain Rel Hydrophobic SASA	Mutated Residue	1
idhSASA_sc_1	Side Chain Rel Hydrophobic SASA	1st Degree Neighbors	1
idhSASA_sc_2	Side Chain Rel Hydrophobic SASA	2nd Degree Neighbors	0
idSASA_0	Solvent Accessible Surface Area	Mutated Residue	1
idSASA_1	Solvent Accessible Surface Area	1st Degree Neighbors	0
idSASA_2	Solvent Accessible Surface Area	2nd Degree Neighbors	0
idSASA_fraction_0	Relative SASA	Mutated Residue	1
idSASA_fraction_1	Relative SASA	1st Degree Neighbors	1
idSASA_fraction_2	Relative SASA	2nd Degree Neighbors	0
idSASA_sc_0	Side Chain SASA	Mutated Residue	1
idSASA_sc_1	Side Chain SASA	1st Degree Neighbors	0
idSASA_sc_2	Side Chain SASA	2nd Degree Neighbors	0
iinterface_hbonds	Total # Hydrogen Bonds	Interface	1
iinterface_residues_0	Residue in Interface	Mutated Residue	0
iinterface_residues_1	Residues in Interface	1st Degree Neighbors	1
iinterface_residues_2	Residues in Interface	2nd Degree Neighbors	0
Ipackstat	Interface Packing Statistics	Interface	0
isc_value	Shape Complementarity	Interface	1
lk_ball_wtd_0	See Rosetta Score Function	Mutated Residue	0
lk_ball_wtd_1	See Rosetta Score Function	1st Degree Neighbors	1
pro_close_0	See Rosetta Score Function	Mutated Residue	0
pro_close_1	See Rosetta Score Function	1st Degree Neighbors	0
sin_if	SIN Scores of Interface Residues	Interface	1
sin_norm	SIN Normalization constant	Full Complex	1
sin_res	SIN Score of mutated residue	Mutated Residue	1

total_score_0	Total Rosetta Energy Score	Mutated Residue	1
total_score_1	Total Rosetta Energy Score	1st Degree Neighbors	1

Table S3. **Overlap of AbRFC and RF Regressor Predicted Mutations in Validation Set.** The predictions of AbRFC and the RF Regressor are highly correlated (spearman r = .88 during cross-validation), but this does not result in a high overlap in the mutations predicted in the top 10,20, or 30% on the validation set. This means that despite the high correlation, the mutations selected in practice by each model would be substantially (>=50%) different.

Top N (%)	# Mutations Predicted by Both AbRFC and RF Regressor	# Mutations Predicted by Either AbRFC or RF Regressor	Overlap Fraction
7(10)	2	12	0.17
15(20)	10	20	0.50
23(30)	16	30	0.53
31(40)	26	36	0.72
39(50)	35	43	0.81

Table S4. VH and VL Sequence Identities Between Datasets. The VH sequence identity between the row and column antibody is in the top half of the matrix and the VL sequence identity is in the bottom half of the matrix. For the row/column denoted "Training," the maximum sequence identity of the antibody to all antibodies in the training set was used. CMAB0 has a maximum VH identity to the training set of 59%, while GMAB0 (S309) has a maximum identity of 74% to the training set, indicating that neither of these antibodies have homologues in the dataset used to train AbRFC.

	**CMAB0**	**GMAB0**	**Training**	**Validation**
**CMAB0**	100	54	59	51
**GMAB0**	63	100	74	46
**Training**	86	84	100	80
**Validation**	46	45	67	100

Table S5. CMAB283 Maintains Binding Advantage over CMAB0 on BA.4/5.

Antibody	BA.1 RBD KD (M)	BA.2 RBD KD (M)	BA.4/5 RBD KD (M)
CMAB0	4.44E-09	9.10E-09	1.91E-08
CMAB283	1.54E-12	2.08E-12	6.02E-12

Supplementary Data

Supplementary Data 1. CMAB0 Saturation Mutagenesis Scores. Refer to the attached excel sheet (**Supplementary Data 1.xls**).

Supplementary Data 2. GMAB0 Saturation Mutagenesis Scores. Refer to the attached excel sheet (**Supplementary Data 2.xls**).

Supplementary Data 3. PDB Sequence Identity and Groups. Refer to the excel sheet (**Supplementary Data 3.xls**)

Supplementary Figures

Figure S1

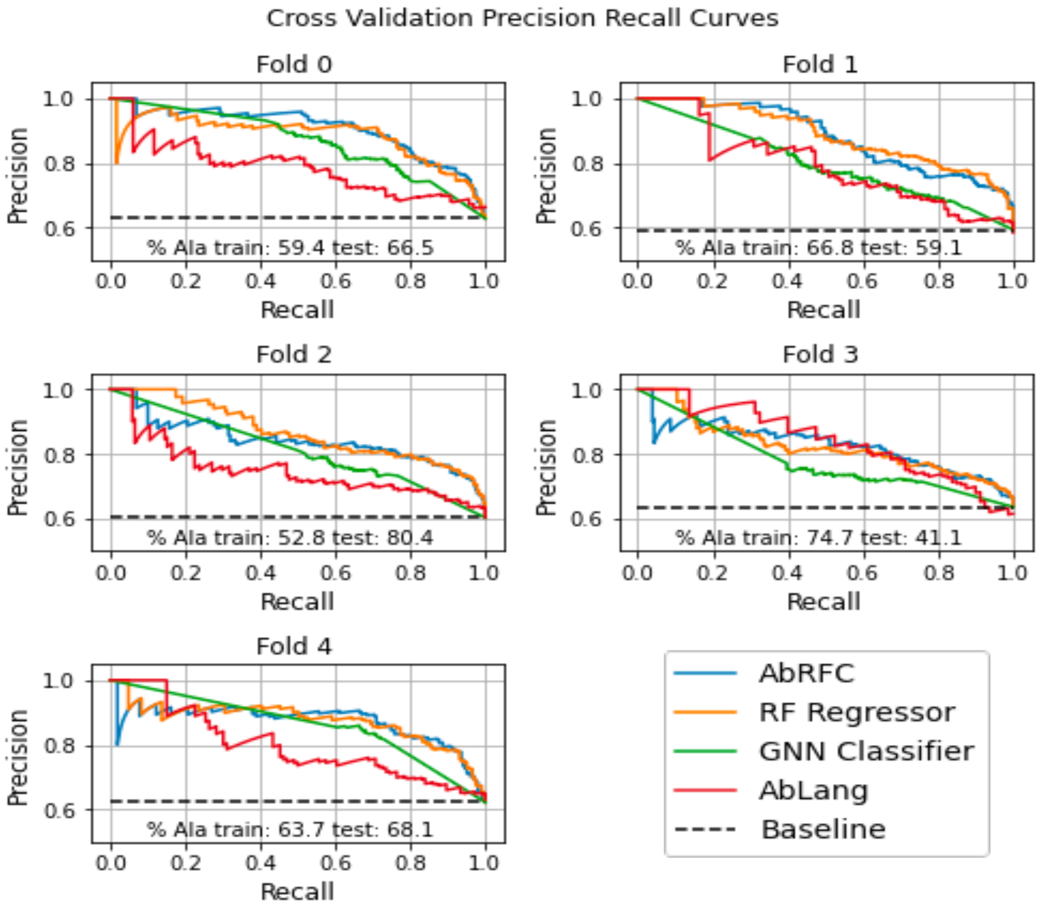

Figure S1. Precision Recall (PR) curves to assess cross-validation performance of the various models. The cutoff used (see methods) results in a random precision of ~.6. All structure-based models show relatively similar performance above baseline, while AbLang performs surprisingly well given that it uses no epitope information and is not trained for this task. Note that the SoftMax scores close to 0 or 1 for the GNN classifier cause a large portion of the PR curve to resemble a straight line.

Figure S2

A.

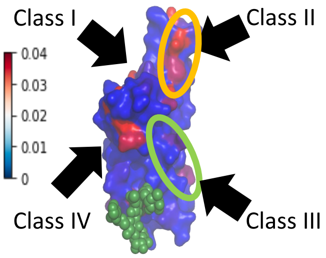

B.

Barnes Class	CoV-AbDab Structure Clonotypes	CoV-AbDab mAbs in Clonotype	Total CoV-AbDab mAbs	OAS Individuals	OAS Sequences	Avg Epitope Entropy
I	31	231	251	67	13835	0.1
II	11	28	64	25	1249	0.1
III	2	4	24	7	65	0.06
IV	5	13	22	22	543	0.03

C.

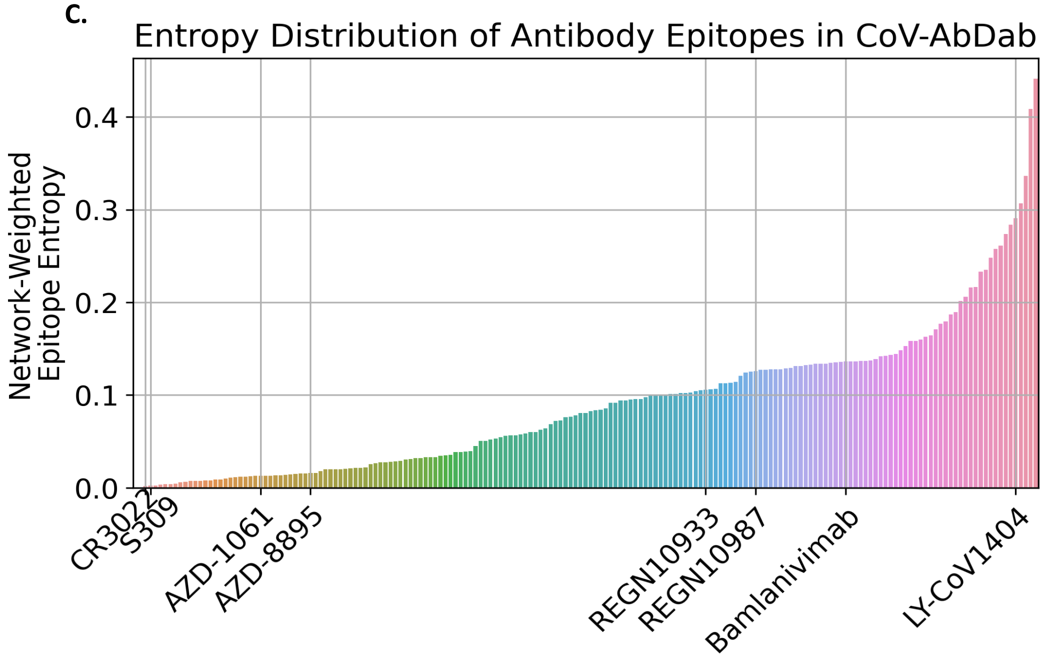

Figure S2. **Selection of template antibodies.** In addition to their low sequence identity to the training set, the templates were also selected for the epitopes they target. **A.** The residue-level entropy in the context of sequence evolution (calculated from GISAID [7] sequences) was used to map the entropy profiles of different epitopes onto the three-dimensional structure of the RBD (shown as surface rendering in purple). The Class III epitope region (encircled in green) encompassing a surface glycosylation (colored green) targeted by antibodies such as S309 and Class IV epitope region targeted by antibodies such as CR3022 have a substantially lower entropy than Class II epitope region targeted by antibodies such as Bamlanivimab (encircled in orange). **B.** Analysis of structures available in CoV-AbDab showed clonotypes. When additional Abs from CoV-AbDab [8] and OAS [9] were mapped to these clonotypes, a bias for ACE2 blocking class I and class II Abs was observed both in the CoV-AbDab subset and the OAS subset. These Ab classes also have, on average, the highest epitope entropies. **C.** Unique class III clonotype S309 and class IV clonotype CR3022 target extremely low-entropy epitopes.

Figure S3

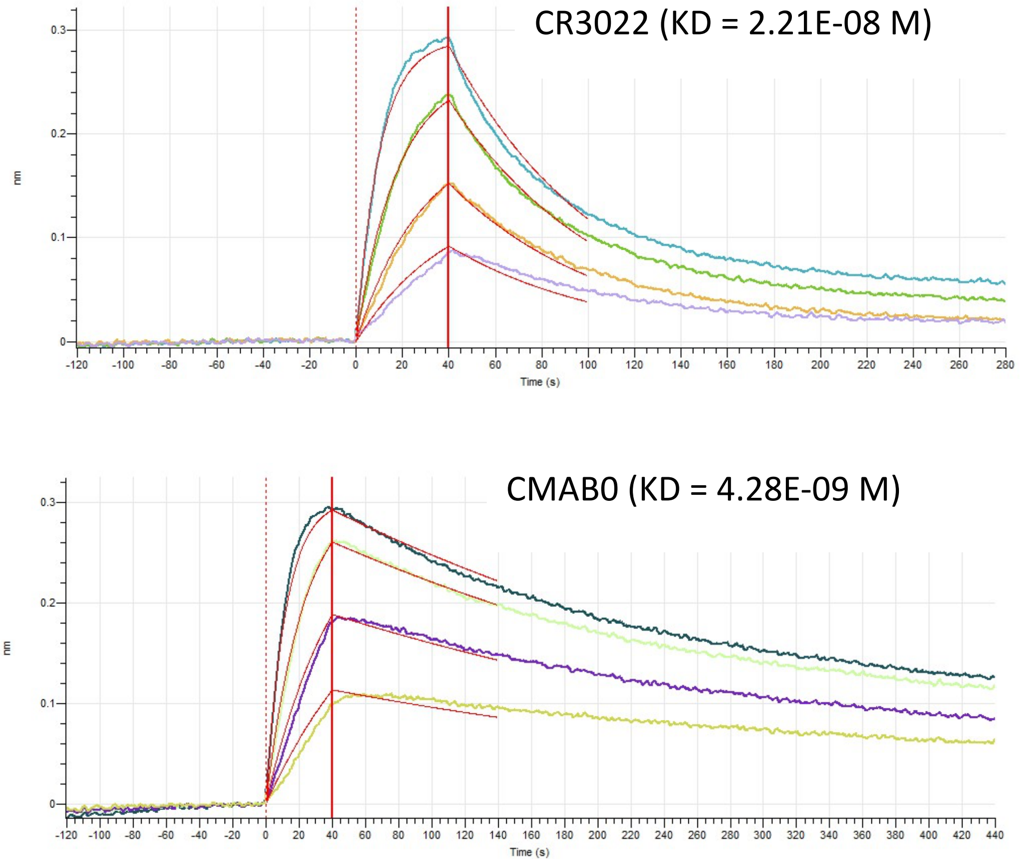

Figure S3. Binding kinetics of CR3022 and CMAB0 against the SARS-COV-2 B.1 (Wuhan) strain measured using biolayer interferometry on the Octet instrument.

Figure S4

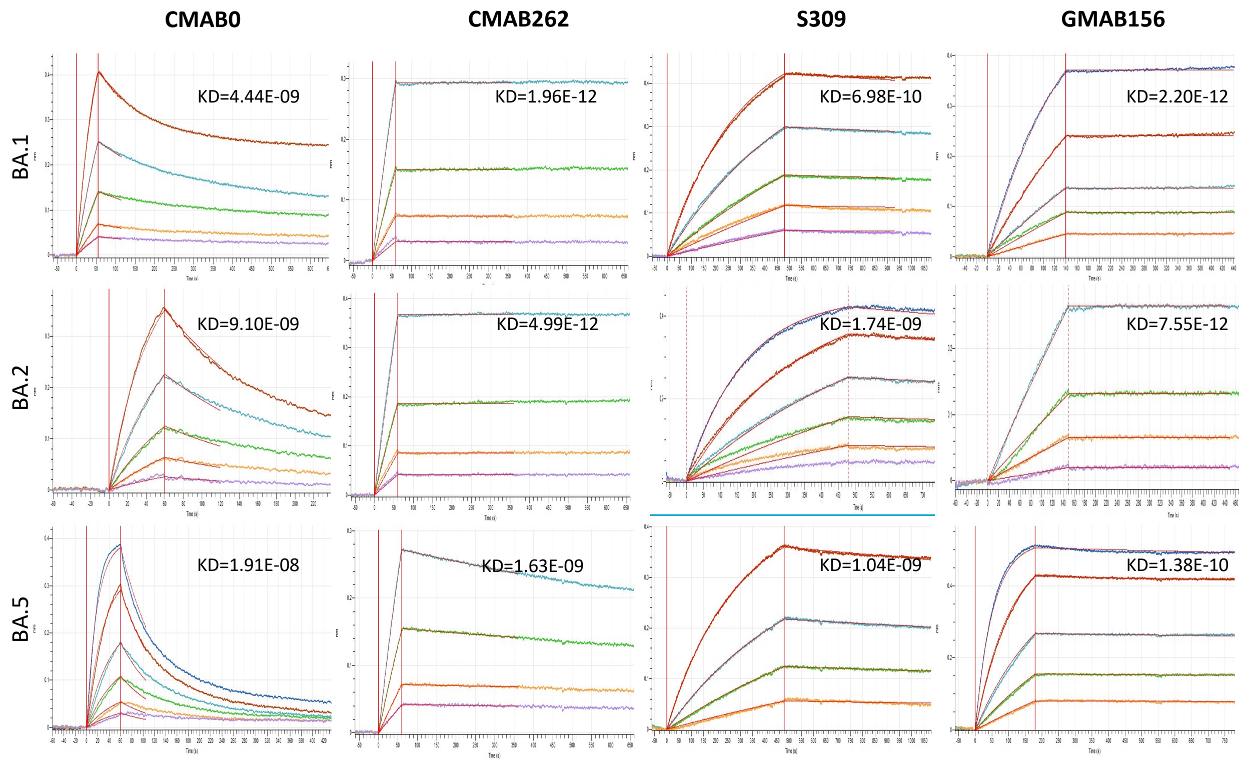

Figure S4. Binding kinetics of CMAB0, S309, CMAB262, and GMAB156 against the SARS-COV-2 Omicron subtypes BA.1, BA.2, and BA.5 measured using biolayer interferometry on the Octet instrument.

Figure S5

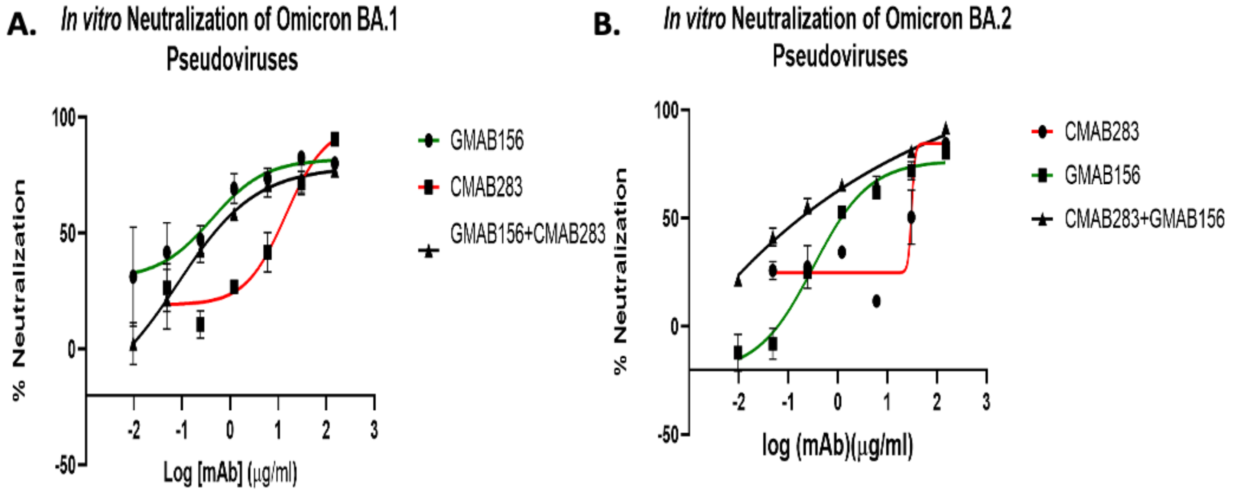

Antibody	IC_{50} (μg/ml)
CMAB283	14.27
GMAB156	0.4116
GMAB156+CMAB283	0.08056

Antibody	IC_{50} (μg/ml)
CMAB283	~30.68
GMAB156	0.3182
GMAB156+CMAB283	<0.2

Figure S5. GMAB156 and CMAB283 show synergistic neutralization against BA.1 and BA.2. Due to its superior binding against BA.4/5 relative to GMAB262, CMAB283 was taken forward with GMAB156 into a pseudovirus neutralization assay (**Supplementary Methods**). The antibodies showed synergistic neutralization, validating targeting orthogonal epitopes with affinity optimized constructs.

Supplementary References

1. Shan, S. et al. Deep learning guided optimization of human antibody against SARS-CoV-2 variants with broad neutralization. *Proc Natl Acad Sci U S A* **119**, e2122954119 (2022).
2. Olsen, T.H., Moal, I.H. & Deane, C.M. AbLang: an antibody language model for completing antibody sequences. *Bioinform Adv* **2**, vbac046 (2022).
3. Liu, X., Luo, Y., Li, P., Song, S. & Peng, J. Deep geometric representations for modeling effects of mutations on protein-protein binding affinity. *PLoS Comput Biol* **17**, e1009284 (2021).
4. Hie, B.L. et al. Efficient evolution of human antibodies from general protein language models. *Nat Biotechnol* (2023).
5. Park, H. et al. Simultaneous Optimization of Biomolecular Energy Functions on Features from Small Molecules and Macromolecules. *J Chem Theory Comput* **12**, 6201-6212 (2016).
6. Chaudhury, S., Lyskov, S. & Gray, J.J. PyRosetta: a script-based interface for implementing molecular modeling algorithms using Rosetta. *Bioinformatics* **26**, 689-691 (2010).
7. Khare, S. et al. GISAID's Role in Pandemic Response. *China CDC Wkly* **3**, 1049-1051 (2021).
8. Raybould, M.I.J., Kovaltsuk, A., Marks, C. & Deane, C.M. CoV-AbDab: the coronavirus antibody database. *Bioinformatics* **37**, 734-735 (2021).
9. Olsen, T.H., Boyles, F. & Deane, C.M. Observed Antibody Space: A diverse database of cleaned, annotated, and translated unpaired and paired antibody sequences. *Protein Sci* **31**, 141-146 (2022).
