# Supplementary material for: Enhancing antibody affinity through experimental sampling of non-deleterious CDR mutations predicted by machine learning
Source: Commun Chem. 2023 Nov 9;6:244. doi: 10.1038/s42004-023-01037-7 (PMC10636138; doi:10.1038/s42004-023-01037-7)
Supplement: Supplementary file 2 — Description of Additional Supplementary Files [file 42004_2023_1037_MOESM2_ESM.pdf]

Description of Additional Supplementary Files

File name: Supplementary Data 1

Description: Scores for saturated mutagenesis on CMAB0

File name: Supplementary Data 2

Description: Scores for saturated mutagenesis on GMAB0

File name: Supplementary Data 3

Description: Sequence Identities and grouping in PDB
