# Supplementary material for: Enhancing antibody affinity through experimental sampling of non-deleterious CDR mutations predicted by machine learning
Source: Commun Chem. 2023 Nov 9;6:244. doi: 10.1038/s42004-023-01037-7 (PMC10636138; doi:10.1038/s42004-023-01037-7)
Supplement: Supplementary file 6 — Reporting Summary [file 42004_2023_1037_MOESM6_ESM.pdf]

nature portfolio

Corresponding author(s): Rahul Raman, Kannan Tharakaraman and Ram Sasisekharan

Last updated by author(s): Aug 17, 2023

Reporting Summary

Nature Portfolio wishes to improve the reproducibility of the work that we publish. This form provides structure for consistency and transparency in reporting. For further information on Nature Portfolio policies, see our Editorial Policies and the Editorial Policy Checklist.

Statistics

For all statistical analyses, confirm that the following items are present in the figure legend, table legend, main text, or Methods section.

n/a	Confirmed	
☒	☐	The exact sample size (*n*) for each experimental group/condition, given as a discrete number and unit of measurement
☒	☐	A statement on whether measurements were taken from distinct samples or whether the same sample was measured repeatedly
☒	☐	The statistical test(s) used AND whether they are one- or two-sided<br>*Only common tests should be described solely by name; describe more complex techniques in the Methods section.*
☒	☐	A description of all covariates tested
☒	☐	A description of any assumptions or corrections, such as tests of normality and adjustment for multiple comparisons
☐	☒	A full description of the statistical parameters including central tendency (e.g. means) or other basic estimates (e.g. regression coefficient) AND variation (e.g. standard deviation) or associated estimates of uncertainty (e.g. confidence intervals)
☒	☐	For null hypothesis testing, the test statistic (e.g. *F*, *t*, *r*) with confidence intervals, effect sizes, degrees of freedom and *P* value noted<br>*Give P values as exact values whenever suitable.*
☒	☐	For Bayesian analysis, information on the choice of priors and Markov chain Monte Carlo settings
☒	☐	For hierarchical and complex designs, identification of the appropriate level for tests and full reporting of outcomes
☐	☒	Estimates of effect sizes (e.g. Cohen's *d*, Pearson's *r*), indicating how they were calculated

Our web collection on statistics for biologists contains articles on many of the points above.

Software and code

Policy information about availability of computer code

Data collection	Plate reader data was collected using Molecular Devices Softmax Pro 6.2.1. Octet data was collected using Sartorius Octet Acquisition 11.1.3.25.
Data analysis	Octet data was analyzed using Sartorius Data Analysis HT. 11.1.3.50. Code to reproduce the central results of the study is available at https://github.com/tbc01/AbRFC.

For manuscripts utilizing custom algorithms or software that are central to the research but not yet described in published literature, software must be made available to editors and reviewers. We strongly encourage code deposition in a community repository (e.g. GitHub). See the Nature Portfolio guidelines for submitting code & software for further information.

Data

Policy information about availability of data

All manuscripts must include a data availability statement. This statement should provide the following information, where applicable:

- Accession codes, unique identifiers, or web links for publicly available datasets
- A description of any restrictions on data availability
- For clinical datasets or third party data, please ensure that the statement adheres to our policy

The original data contributions of this article are included in the article/Supplementary Materials. The publicly available data used in this study are available as follows. The collated dataset of experimental data used to train and evaluate AbRFC and other models is available from the mcsm-Ab2 website (https://

biosig.lab.uq.edu.au/mcsm_ab2/data). All PDBs for this dataset, the validation dataset, and for the 2 SARS-COV-2 test examples were downloaded from the Protein Data Bank (https://rcsb.org). The code and relevant data to reproduce the results in this study is available at https://github.com/tbc01/AbRFC.

Research involving human participants, their data, or biological material

Policy information about studies with human participants or human data. See also policy information about sex, gender (identity/presentation), and sexual orientation and race, ethnicity and racism.

Reporting on sex and gender	*Use the terms sex (biological attribute) and gender (shaped by social and cultural circumstances) carefully in order to avoid confusing both terms. Indicate if findings apply to only one sex or gender; describe whether sex and gender were considered in study design; whether sex and/or gender was determined based on self-reporting or assigned and methods used.*<br>*Provide in the source data disaggregated sex and gender data, where this information has been collected, and if consent has been obtained for sharing of individual-level data; provide overall numbers in this Reporting Summary. Please state if this information has not been collected.*<br>*Report sex- and gender-based analyses where performed, justify reasons for lack of sex- and gender-based analysis.*
Reporting on race, ethnicity, or other socially relevant groupings	*Please specify the socially constructed or socially relevant categorization variable(s) used in your manuscript and explain why they were used. Please note that such variables should not be used as proxies for other socially constructed/relevant variables (for example, race or ethnicity should not be used as a proxy for socioeconomic status).*<br>*Provide clear definitions of the relevant terms used, how they were provided (by the participants/respondents, the researchers, or third parties), and the method(s) used to classify people into the different categories (e.g. self-report, census or administrative data, social media data, etc.)*<br>*Please provide details about how you controlled for confounding variables in your analyses.*
Population characteristics	*Describe the covariate-relevant population characteristics of the human research participants (e.g. age, genotypic information, past and current diagnosis and treatment categories). If you filled out the behavioural & social sciences study design questions and have nothing to add here, write "See above."*
Recruitment	*Describe how participants were recruited. Outline any potential self-selection bias or other biases that may be present and how these are likely to impact results.*
Ethics oversight	*Identify the organization(s) that approved the study protocol.*

Note that full information on the approval of the study protocol must also be provided in the manuscript.

Field-specific reporting

Please select the one below that is the best fit for your research. If you are not sure, read the appropriate sections before making your selection.

☒ Life sciences ☐ Behavioural & social sciences ☐ Ecological, evolutionary & environmental sciences

For a reference copy of the document with all sections, see nature.com/documents/nr-reporting-summary-flat.pdf

Life sciences study design

All studies must disclose on these points even when the disclosure is negative.

Sample size	Sample size for the final binding kinetics readout was determined to be N>=2 biological replicates (transfections) per construct based to ensure reproducibility of the data.
Data exclusions	No data was excluded in this study.
Replication	N>=2 biological replicates were run on the Octet to ensure the reproducibility of the binding kinetic numbers.
Randomization	This is not relevant to the study.
Blinding	All researchers performing the experiments were blinded to the data.

Reporting for specific materials, systems and methods

We require information from authors about some types of materials, experimental systems and methods used in many studies. Here, indicate whether each material, system or method listed is relevant to your study. If you are not sure if a list item applies to your research, read the appropriate section before selecting a response.

Materials & experimental systems

n/a	Involved in the study
☐	☒ Antibodies
☐	☒ Eukaryotic cell lines
☒	☐ Palaeontology and archaeology
☒	☐ Animals and other organisms
☒	☐ Clinical data
☒	☐ Dual use research of concern
☒	☐ Plants

Methods

n/a	Involved in the study
☒	☐ ChIP-seq
☒	☐ Flow cytometry
☒	☐ MRI-based neuroimaging

Antibodies

Antibodies used

Template and engineered antbodies were made in-house. Secondary antibody used in ELISA HRP-labeled rabbit anti-human antibody

Validation

Where applicable, antibodies were sequence verified and tested for antigen binding across at least 2 lots.

Eukaryotic cell lines

Policy information about cell lines and Sex and Gender in Research

Cell line source(s)

ExpiCHO and Expi293 cells (antibody production) and HEK293T cell lines stably expressing human ACE2 and human TMPRSS2 (for pseudovirus neutralization)

Authentication

HEK293T: https://www.genecopoeia.com/wp-content/uploads/2020/08/Datasheet-SL222-HEK293T-ACE2TMPRSS2.pdf
ExpiCHO: https://www.thermofisher.com/order/catalog/product/A29127?SID=srch-srp-A29127
Expi293: https://www.thermofisher.com/order/catalog/product/A14527?SID=srch-srp-A14527

Mycoplasma contamination

Vendors COA indicates cell line is free of mycoplasma

Commonly misidentified lines
(See ICLAC register)

Name any commonly misidentified cell lines used in the study and provide a rationale for their use.
